# The role of disulfidptosis-driven tumor microenvironment remodeling in pancreatic cancer progression

**DOI:** 10.3389/fimmu.2026.1747560

**Published:** 2026-03-10

**Authors:** Wenhao Li, Jiewen Deng, Hao Zhang, Menghan Zhang, Chuanxu Kang, Zicheng Yu, Yunshen Gu, Xiaobo He, Yanfeng Wu

**Affiliations:** 1 National Key Laboratory of Immunity and Inflammation & Institute of Immunology, College of Basic Medical Sciences, Naval Medical University, Shanghai, China; 2 Department of Cardiovascular Diseases, Ninth People’s Hospital, Shanghai Jiao Tong University School of Medicine, Shanghai, China

**Keywords:** disulfidptosis, immune infiltration, pancreatic cancer, prognosis, tumor microenvironment, UBASH3B

## Abstract

**Background:**

Pancreatic ductal adenocarcinoma (PDAC) continues to pose a significant clinical challenge due to its high mortality rate and limited treatment efficacy. The role of disulfidptosis, a recently discovered mode of regulatory cell death, in pancreatic cancer progression and tumor immunity remains poorly understood.

**Methods:**

We developed and validated a disulfidptosis-related prognostic signature based on 13 pivotal genes. Integrated multi-omics approaches, incorporating bulk and single-cell transcriptomics, spatial transcriptomics, and functional assays, were employed to investigate the association between disulfidptosis and key biological processes.

**Results:**

The prognostic signature effectively stratified PDAC patients into distinct risk categories with markedly different clinical outcomes and exhibited consistent predictive accuracy across multiple independent cohorts. It informed personalized treatment strategies, revealing differential sensitivity to therapeutic agents including dasatinib and UMI-77. Multi-omics analyses revealed that disulfidptosis is closely associated with redox homeostasis, cytoskeletal organization, and immune evasion. The high-risk subgroup displayed an immunosuppressive tumor microenvironment, characterized by diminished CD8^+^ T cell infiltration and altered immune checkpoint expression. UBASH3B was further identified as a core prognostic biomarker, significantly promoting the proliferation and migration of pancreatic cancer cells, while bioinformatic analysis also suggested its association with tumor immune regulation pathway.

**Conclusion:**

Our study develops a novel prognostic framework for patient stratification based on disulfidptosis-related genes in PDAC. Integrated multi-omics analyses reveal a strong association between disulfidptosis and an immunosuppressive tumor microenvironment, positioning it as a candidate metabolic vulnerability that warrants further functional investigation. These findings offer correlative insights into the involvement of disulfidptosis in immune regulation and suggest that further exploration of this pathway may inform future immunotherapeutic strategies.

## 1 Introduction

Pancreatic ductal adenocarcinoma (PDAC), the most common and highly aggressive form of pancreatic cancer, has a dismal 5-year survival rate of approximately 10%. This poor prognosis is primarily attributed to challenges in early detection, a high propensity for metastasis, and resistance to conventional therapies (1–3). Emerging evidence has highlighted a novel form of regulated cell death, disulfidptosis, which is intrinsically linked to cellular metabolic state. Its core mechanism involves: under conditions of glucose deprivation, cells with high expression of the cystine transporter solute carrier family 7-member 11 (SLC7A11) undergo excessive cystine uptake. Concomitantly, impaired NADPH regeneration leads to the depletion of reducing capacity, resulting in the aberrant accumulation of disulfide molecules. These disulfides subsequently induce the formation of abnormal disulfide bonds among actin cytoskeleton proteins, culminating in cytoskeletal network collapse and cell death (4).

Recent studies have established a relatively comprehensive understanding of disulfidptosis, linking it directly to key tumor characteristics, including progression, metastasis and therapy resistance (5–8). Drivers like SLC7A11 and FLNA are frequently altered via mutations, copy−number variations, or promoter hypomethylation, priming tumors for disulfidptosis susceptibility, as observed in pan−cancer analyses (6, 7). Furthermore, disulfide stress directly cross−links actin, FLNA, and tubulin, leading to fatal network collapse, a key mechanism reported in colorectal cancer (6); this process is dynamically regulated by proteins such as INF2, which coordinates mitochondrial fission and actin remodeling, as highlighted in ovarian cancer research (7). Metabolically, the pathway engages with glucose transport, NADPH regeneration, and oxidative phosphorylation, while intersecting with oncogenic signaling cascades including TGF−β and Wnt across various cancers (5, 7). This form of cell death also crosstalk with cell−cycle progression and DNA−repair pathways, thereby influencing responses to chemotherapy, PARP inhibitors, and immunotherapy (8). However, the specific role of disulfidptosis in the pathogenesis of pancreatic cancer remains poorly defined. Although preliminary evidence suggests a potential regulatory function of disulfidptosis in pancreatic cancer progression (9, 10), a more systematic and in-depth investigation is still lacking. Notably, the tumor microenvironment (TME) of PDAC is characterized by severe desmoplasia, extensive fibrosis, and vascular paucity, leading to profound nutrient deprivation and hypoxia (11–13). Hypoxia-induced redox imbalance and glucose deprivation create conditions conducive to disulfidptosis. Furthermore, as an inflammation-driven malignancy, chronic stimulation by inflammatory cytokines subjects various cells within the TME to persistent stress (12), which may further favor the occurrence of disulfidptosis. A recent study demonstrated that antioxidant treatment can protect pancreatic cancer cells from excessive disulfide accumulation under glucose starvation and that restoring the pentose phosphate pathway activity helps stabilize NADPH levels to inhibit disulfidptosis, thereby providing the first direct experimental evidence for the existence of this cell death pathway in PDAC (9). This pivotal finding not only confirms the susceptibility of PDAC to disulfidptosis but also prompts a deeper investigation into the specific cellular regulators that govern susceptibility to disulfidptosis in pancreatic cancer.

Regulated cell death (RCD) modulates immune and stromal cells in the dual-natured way: mild apoptosis restrains growth, but overwhelming death under stress releases immunosuppressive mediators and pro-oxidant lipids that erase effector lymphocytes—ferroptosis being one example (14–16). This mechanism may partly explain the frequently observed low infiltration or exhausted state of effector T cells in pancreatic cancer. Recent studies indicate that disulfidptosis can lead to CD8^+^T cell exhaustion and promote cancer progression (17). This study systematically analyses the expression patterns of disulfidptosis-related genes in pancreatic cancer, investigating their association with TME remodeling. It also leads to the development of a disulfideptosis-related prognostic model. UBASH3B was identified as a prognostic biomarker that is significantly correlated with patient survival. Further analysis suggests that UBASH3B may be associated with tumor immune responses and malignant phenotypic transition, potentially through its involvement in pancreatic cancer cell responses to TME-related factors, thereby promoting disease progression. These findings provide a molecular basis for understanding the role of disulfidptosis in the immunoregulation of pancreatic cancer. The established prognostic model provides a theoretical basis for developing early diagnostic markers and personalized treatment strategies, demonstrating its potential clinical value in advancing precision immunotherapy for pancreatic cancer.

This study aims to construct a disulfidptosis-related prognostic model for pancreatic cancer, systematically characterize its correlations with the tumor immune microenvironment, and identify relevant driver genes and potential therapeutic targets. By integrating multi-omics data, we have identified UBASH3B as an independent prognostic biomarker and preliminarily suggested a potential correlation for UBASH3B in driving pancreatic cancer progression. These findings not only deepen the understanding of disulfidptosis in pancreatic cancer but also provide a theoretical foundation and potential targets for developing early diagnostic markers and precision immunotherapy strategies.

## 2 Materials and methods

### 2.1 Data collection and preprocessing

**Figure 1:**
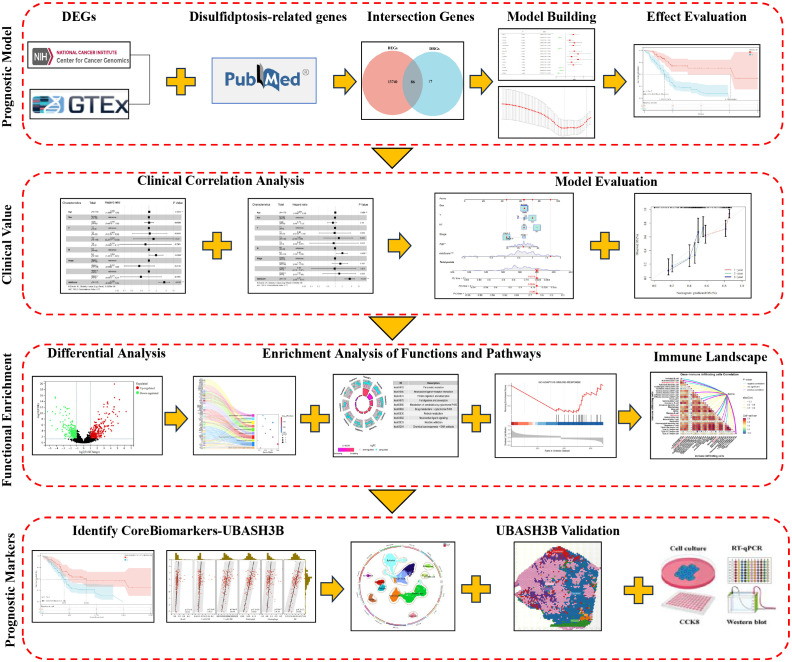
Workflow graph.

**Figure 1** shows the specific process of all the work in this study. Transcriptomic and clinical data were assembled from public repositories. RNA sequencing (RNA-seq) data from PAAD samples came from The Cancer Genome Atlas (TCGA), which included 177 tumor and 4 normal tissue specimens. An additional 167 normal pancreatic tissue samples were sourced from the Genotype-Tissue Expression (GTEx) database to augment the normal tissue cohort for comparative analysis. The accompanying clinical dataset from TCGA-PAAD contained demographic and clinicopathological variables (gender, age, tumor stage) as well as survival outcomes (survival time and status). Furthermore, we procured three independent datasets—GSE28735, GSE62452, and GSE78229—from the Gene Expression Omnibus (GEO) to serve as external validation cohorts for prognostic model evaluation. Samples lacking complete survival information were omitted from the analysis. A curated list of disulfidptosis-related genes (DRGs) was acquired from an established publication (4) (see **Supplementary Table 1** for the full list).

### 2.2 Identification of DEGs

Differential expression analysis between tumor and normal tissues was performed using the R package DESeq2 on HTseq-Counts data. Genes with an absolute log2 fold change (|log2FC|) > 1 and an adjusted p-value< 0.05 were defined as statistically significant differentially expressed genes (DEGs) for pancreatic cancer. The same method was subsequently applied to identify DEGs between high- and low-risk groups, using a threshold of |log2FC| > 2 and adjusted p-value< 0.05. The overlap between pancreatic cancer DEGs and DRGs was identified using a Venn diagram for subsequent analyses.

### 2.3 Construction and validation of the DRG prognostic model

Univariate Cox regression analysis was performed on the overlapping genes using the coxph function from the R survival package to identify genes significantly associated with prognosis (p< 0.05). The resulting significant genes were then subjected to least absolute shrinkage and selection operator (LASSO) Cox regression analysis using the glmnet package to refine the gene signature and prevent overfitting (18). The optimal penalty parameter (λ) was determined via 10-fold cross-validation using the cv.glmnet function. The λ value that yielded the minimum cross-validated error (λ_min = 0.044) was selected to construct the final prognostic model, ensuring the best predictive performance. Genes retained at the λ_min, along with their coefficients, were used to calculate a risk score for each pancreatic cancer patient in the TCGA-PAAD cohort according to the formula:


$$Risk\;score\;=\sum_{n}^{i}x_{i}y_{i}$$

The optimal cutoff value for the risk score was determined using the maxstat R package, with group sizes constrained between 25% and 75% of the cohort. Patients were stratified into high- and low-risk groups based on this cutoff. The prognostic performance of the model was evaluated by comparing overall survival (OS) between the two risk groups using Kaplan-Meier (K-M) analysis (19). Time-dependent receiver operating characteristic (timeROC) analysis and ROC analysis were conducted using the pROC package to assess the model’s predictive accuracy, with the ci function used to determine the area under the ROC curve (AUC) and its confidence interval (20). For external validation, the model’s prognostic value was further tested in three independent GEO datasets (GSE28735, GSE62452, and GSE78229) using K-M survival analysis, timeROC, and ROC analysis. All gene expression data were normalized and standardized prior to analysis. We utilized shapely additive explanations (SHAP) to quantify the contribution of individual features to the model’s output. This method provides a detailed understanding of the magnitude and direction of each variable’s influence on the final prediction. To explore the functional interactions and regulatory context of the model genes, we conducted a PPI network analysis via STRING, followed by an interrogation of upstream transcription factors and downstream miRNA regulators using NetworkAnalyst.

### 2.4 Functional enrichment and GSEA

TCGA-PAAD patients were stratified into high- and low-risk subgroups based on the median disulfidptosis risk score. DEGs between these subgroups were identified. Functional enrichment analysis of these DEGs was performed using the clusterProfiler R package for Gene Ontology (GO) and Kyoto Encyclopedia of Genes and Genomes (KEGG) pathways (21). Terms with an adjusted p-value< 0.05 and a gene count > 2 were considered significantly enriched. The top 20 most significant terms were selected for visualization. Additionally, Gene Set Enrichment Analysis (GSEA) was employed to uncover subtle but coordinated pathway-level differences between the risk subgroups (22). Significantly enriched pathways were defined as those with a p-value< 0.05 and an absolute normalized enrichment score > 1.

### 2.5 Analysis of single-cell RNA sequencing

The pancreatic cancer single-cell RNA sequencing (scRNA-seq) dataset GSE155698 was obtained from the GEO database, comprising 16 tumor and 3 normal samples. Data preprocessing and analysis were conducted using the Seurat R package. Initial quality control involved filtering out genes detected in fewer than 3 cells and cells expressing fewer than 250 genes. Cells with a mitochondrial gene ratio exceeding 15% or an erythrocyte gene ratio above 3% were excluded. The remaining data were normalized using the NormalizeData function. The top 3000 highly variable genes were identified with FindVariable and scaled using ScaleData. Batch effects across the 19 samples were corrected using Harmony, followed by dimensionality reduction via UMAP (23). Cells were clustered at a resolution of 0.3 and annotated into distinct cell types based on established canonical markers (24). The proportional abundance and gene expression profiles of each cell type across different tissues were subsequently analyzed.

To evaluate disulfidptosis activity, a disulfidptosis score was calculated for each cell using the AUCell_calcAUC function from the AUCell package, based on the prognostic DRGs (25). Cells were then stratified into high- and low-score groups based on this metric. Similarly, to investigate the role of UBASH3B in the tumor microenvironment, epithelial cells were dichotomized into high- and low-expression subgroups according to UBASH3B expression levels. Cell-cell communication networks were inferred using the CellChat package to characterize interactions mediated by human ligand-receptor pairs (26). The strength and number of interactions were compared between the different disulfidptosis score groups and UBASH3B expression subgroups, with significant ligand-receptor pairs identified using the Wilcoxon rank-sum test (p< 0.05). DEGs between the high and low disulfidptosis score groups, as well as between UBASH3B high- and low-expressing epithelial cells, were identified using the FindMarkers function. DEGs were defined as those with an absolute log2 fold change |log2FC| > 0.25, a p-value< 0.05, and expressed in more than 10% of cells within either group. GO and KEGG enrichment analyses were performed on these DEGs to uncover significant functional pathways (p< 0.05). GSEA was further employed to investigate the enrichment of relevant signaling pathways among the DEGs.

### 2.6 Analysis of spatial transcriptomics data

For spatial transcriptomics analysis, dataset GSE277783 was downloaded from GEO, and sample GSM8452856 was selected for in-depth study. The data were processed using the Seurat package, normalized via the SCTransform method, and the most variable features were identified (27). Unsupervised clustering was performed following dimensionality reduction with RunPCA. Cell type annotation was transferred from the scRNA-seq analysis based on canonical markers. The spatial expression patterns of key genes, including UBASH3B, were visualized using SpatialFeaturePlot. The disulfidptosis score was also calculated and visualized across spatial locations using a method analogous to the scRNA-seq analysis. This integrated approach facilitated the investigation of spatial heterogeneity in key gene expression and regional variations in disulfidptosis activity within the pancreatic cancer tissue.

### 2.7 Clinical translation and genomic characterization of the prognostic DRGs

To evaluate the clinical relevance of the DRG signature, we first assessed its potential as an independent prognostic factor. Univariate and multivariate Cox regression analyses were performed to determine the association between the risk score and overall survival, after adjusting for other key clinical-pathological variables (e.g., age, sex, tumor stage). Subsequently, a comprehensive nomogram was constructed by integrating the risk score with these significant clinical parameters using the rms package in R (28). This nomogram serves as a quantitative tool to predict the probability of individual patient survival at specific time points (e.g., 1, 2, 3 years). The predictive accuracy and calibration of the nomogram were visually assessed using calibration curves, which compare the predicted probabilities against the actual observed outcomes. The clinical applicability of the predictive models was assessed using Decision Curve Analysis (DCA). This analysis compared the net benefit of the nomogram, the risk score, and relevant clinical variables against the ‘treat-all’ and ‘treat-none’ strategies over a spectrum of clinically plausible threshold probabilities.

To explore the genomic context of the DRG signature, we analyzed tumor mutational burden, defined as the total number of somatic mutations per megabase of DNA sequenced (29). Using somatic mutation data from the TCGA-PAAD cohort, we investigated the mutational landscape of the prognostic DRGs. Furthermore, we compared the overall tumor mutational burden and the specific mutation profiles of key cancer-associated genes between the high-risk and low-risk patient groups defined by our model.

### 2.8 Analysis of tumor immune microenvironment and immune landscape

The ESTIMATE algorithm was applied to infer the tumor microenvironment composition, generating stromal, immune, and ESTIMATE scores, as well as tumor purity for each patient. Wilcoxon test was used to compare these scores between the risk subgroups. Furthermore, the expression levels of a panel of classical and emerging immune checkpoint genes, curated from published literature, were analyzed for differential expression between the groups (30). The relative abundances of 22 immune cell subtypes within the tumor microenvironment were quantified using the CIBERSORT deconvolution algorithm (31). Differences in immune cell infiltration proportions between high- and low-risk patients were assessed using the Wilcoxon test. Simultaneously, single-sample Gene Set Enrichment Analysis (ssGSEA) was implemented via the GSVA package to quantify the infiltration levels of 28 immune cell types based on their respective gene signatures (32). This method was also applied to evaluate the activity of various immune-related functional pathways. Wilcoxon test was used to determine significant differences in both immune cell infiltration and pathway activity between the two risk groups. The CIDE website (https://cide.ccr.cancer.gov/) was employed to determine the expression profile of UBASH3B across different immune cell types. The TIP website (https://biocc.hrbmu.edu.cn/TIP) was used to analyze the strength of anti-tumor immune responses at different stages in cancer patients. The immunotherapy cohorts utilized in this study included IMvigor210, GSE100797, GSE165252, GSE78220, and GSE163839.

### 2.9 Identification of immune score-associated modules and hub genes via WGCNA

Weighted Gene Co-expression Network Analysis (WGCNA) was performed using the R package of the same name to identify gene modules significantly associated with specific clinical traits (33). The analysis was conducted on DEGs derived from the high- and low-risk subgroups. A soft-thresholding power of β = 4 was selected to ensure a scale-free network topology, resulting in the identification of 8 distinct co-expression modules. Module-trait relationships were assessed by calculating the correlation between each module’s eigengene and the four scores generated by the ESTIMATE algorithm (stromal, immune, ESTIMATE, and tumor purity). The module demonstrating the strongest and most significant correlation with the immune-related traits was selected for further investigation. Genes within the key module were subsequently subjected to protein-protein interaction network analysis using the STRING database. The resulting network was imported into Cytoscape, and the top 10 hub genes were identified using the Maximal clique centrality algorithm via the cytoHubba plugin. Finally, KEGG pathway enrichment and immune-related functional analyses were performed on these hub genes to elucidate their potential roles in mediating the immune functional disparities observed between the high- and low-risk patient subgroups.

### 2.10 Refinement and validation of prognostic biomarkers

To further refine the prognostic gene signature, we employed two distinct machine learning approaches: Random Forests (RF) and Support Vector Machine-Recursive Feature Elimination (SVM-RFE). The SVM-RFE algorithm, implemented using the R package e1071, was used to rank genes based on their relative importance through recursive feature elimination (34). Concurrently, the RF algorithm, executed with the RandomForest R package, provided an independent ranking of genes, leveraging its recognized strength in selecting relevant features and mitigating redundancy compared to linear discriminant analysis or mean squared error methods (35). Genes consistently prioritized by both machine learning algorithms, and further supported by survival analysis and immune infiltration scores from TIMER, were selected as candidate prognostic biomarkers. The expression patterns of the final candidate gene were systematically examined across multiple cancer types and corresponding normal tissues using the Sangerbox platform (36). Furthermore, we assessed its relationship with the tumor microenvironment by evaluating correlations between the candidate gene expression and stromal, immune, and ESTIMATE scores. To independently validate the differential expression of the key candidate gene UBASH3B in pancreatic cancer, we analyzed three external transcriptomic datasets (GSE28735, GSE62452, and GSE71229), comparing its expression levels between tumor and normal pancreatic tissues.

### 2.11 Analysis of drug sensitivity differences between different subgroups

To investigate potential differences in therapeutic response, we employed the R package oncoPredict to calculate the drug sensitivity (as quantified by the estimated IC50) for each pancreatic cancer patient in the cohort (37). Differences in drug sensitivity profiles between the predefined high-risk and low-risk groups, as well as between subgroups stratified by the expression level of the key prognostic biomarker, were assessed using the Wilcoxon rank-sum test. Compounds with a statistically significant difference in sensitivity (p< 0.05) between the compared groups were identified as candidate agents with potential differential therapeutic efficacy. The structures of the small-molecule drugs were obtained from PubChem, and the protein structure of UBASH3B was retrieved from the RCSB PDB database (https://www.rcsb.org). Energy minimization for the small molecules was performed using Chem3D software. The determination of the docking grid and the execution of molecular docking were completed using AutoDock software. PyMOL software was used for visualization of the molecular docking results.

### 2.12 InferCNV infers malignancy of epithelial cells

Copy number variation (CNV) inference was performed using inferCNV (v1.16.0) to assess chromosomal instability and stratify epithelial cells by malignancy grade. Normal cells served as a reference baseline. Gene expression counts from epithelial cells were normalized and smoothed against this reference, and large-scale chromosomal gains or losses were subsequently inferred via a hidden Markov model. Cells were classified into discrete malignancy states based on the cumulative burden and pattern of CNVs.

### 2.13 Cellular trajectory reconstruction analysis

Cellular stemness and differentiation potential were quantified using the CytoTRACE algorithm, which estimates a cell’s developmental state based on its transcriptional diversity—a metric inversely correlated with differentiation. The CytoTRACE score was computed for each cell from its unspliced transcript counts, with higher scores indicating greater stemness and less differentiation. Analyses were conducted using default parameters.

### 2.14 Single-cell pseudotime analysis

Pseudotemporal trajectory analysis was performed using the Monocle2 R package (v.2.26.0) to model cellular state transitions within the epithelial compartment. Cells exhibiting low differentiation potential, as identified by CytoTRACE, were designated as the trajectory root. A CellDataSet object was constructed using a negative binomial expression family. Dimensionality reduction was carried out with the reduce dimension function, and the trajectory was visualized with plot_cell_trajectory. Genes displaying significant variation along pseudotime were identified using differentialGeneTest and subsequently clustered into expression modules via plot_pseudotime_heatmap.

### 2.15 Cell culture

The human pancreatic ductal epithelial cell line HPNE and the human pancreatic cancer cell lines PANC-1, BXPC-3, CFPAC-1, and SW1990, preserved in our laboratory, were used in this study. All cell lines were cultured in DMEM/F-12 medium (Gibco, 11330032) supplemented with 10% FBS (Gibco, 10099141C) and maintained in a humidified incubator at 37 °C with 5% CO_2_. Cells were passaged when they reached 80–90% confluence. All subsequent experiments were performed using cells in the logarithmic growth phase.

### 2.16 Quantitative real-time PCR

Total RNA was extracted from cells using an RNA extraction kit (Fastagen, 220011). The extracted RNA was reverse-transcribed into cDNA using a reverse transcription kit (Takara, RR036A). Quantitative real-time PCR analysis was then performed using TB Green^®^ Premix Ex Taq™ (Takara, RR820A) on a QuantStudio 6 Flex system. The expression levels of target genes were normalized to that of β-actin. The primer sequences were as follows:

β-actin: F: 5’-TGGCACCCAGCACAATGAA-3’;

R: 5’-CTAAGTCATAGTCCGCCTAGAAGCA-3’;

UBASH3B: F: 5’-TGGTTTCCGAGATTACGAGAAA-3’;

R: 5’-TTTTGTCCACTCAAATAAGCCG-3’.

### 2.17 Protein expression analysis of UBASH3B

The protein expression pattern of UBASH3B in normal human pancreatic tissue was retrieved from the Human Protein Atlas database (https://www.proteinatlas.org/). Immunohistochemistry images demonstrating UBASH3B staining in normal pancreatic tissue sections were downloaded and qualitatively analyzed for expression levels and subcellular localization.

Western blot analysis was performed to validate UBASH3B protein expression in pancreatic cancer cells. Total protein was extracted from cells using M-PER Mammalian Protein Extraction Reagent (Thermo Fisher, 78501). Twenty micrograms of protein per sample were separated by electrophoresis on 4–20% SurePAGE gels (GenScript, M00657). The separated proteins were then transferred onto PVDF membranes using the eBlot L1 transfer system (GenScript, China). The membranes were blocked for 2 hours at room temperature with Tris-buffered saline containing 0.1% Tween-20 (TBST) and 5% non-fat dry milk. Subsequently, the membranes were incubated overnight at 4 °C with a UBASH3B polyclonal antibody (Thermo Fisher, 19563-1-AP) diluted at 1:1000. After washing with TBST, the membranes were incubated for 1 hour with an HRP-linked secondary antibody (Cell Signaling Technology, 7074P2) diluted at 1:1000. Protein bands were visualized using an ECL detection reagent (Thermo Scientific, 34095).

### 2.18 RNA interference

Cells were transfected with 10 µM small interfering RNA (siRNA) using the RNAiMAX transfection reagent (Thermo Fisher, 13778030). After 6 hours of transfection, the medium containing the transfection reagent and siRNA was removed and replaced with fresh normal culture medium. The siRNAs were purchased from Tsingke Biotechnology (Shanghai, China). The siRNA sequences were as follows:

siUBASH3B: 5’-GUCAGUCGCUGGAAAUGUA-3’

### 2.19 CCK-8

Cell proliferation was assessed using the Cell Counting Kit-8 (Dojindo, CK04). Briefly, cells were seeded in 96-well plates at a density of 2×10³ cells per well and allowed to adhere overnight. The cells were then transfected as described previously. At 48 hours post-transfection, 10 µL of CCK-8 reagent was added to each well, followed by incubation at 37 °C for 2 hours. The absorbance at a wavelength of 450 nm was measured using the Tecan Spark multimode microplate reader (Tecan, Switzerland).

### 2.20 Wound healing assay

Transfected cells were seeded into 6-well plates. After overnight adherence, a uniform scratch wound was created in the cell monolayer using a sterile plastic pipette tip. The detached cells were gently washed away with PBS, and fresh serum-free medium was added. The wound gaps were imaged at 0, 6, 12, and 24 hours using the Celloger Mini Plus live cell imaging system (Curiosis, Korea). The migration area was quantified by measuring the changes in the scratch area using ImageJ software.

### 2.21 Statistical analysis

All statistical analyses were performed using R software (version 4.4.0) and GraphPad Prism (version 9.0). Data are presented as mean ± standard deviation from at least three independent experiments. For comparisons between two groups, Student’s t-test or Wilcoxon rank-sum test was applied based on data distribution normality. Multiple group comparisons were analyzed using one-way ANOVA with Tukey’s *post-hoc* test. Survival analysis was conducted using Kaplan-Meier curves with log-rank test. Correlation analyses were performed using Pearson or Spearman methods depending on data distribution characteristics. A two-tailed p-value< 0.05 was considered statistically significant. For multiple testing corrections, false discovery rate adjustment was applied where appropriate. Specific statistical methods for each experiment are detailed in the corresponding figure legends.

## 3 Result

### 3.1 Disulfidptosis-related alterations in pancreatic cancer tissues

Gene expression analysis between tumor and normal tissues was performed using the DESeq2 method on datasets from TCGA and GTEx. A total of 1755 upregulated genes and 918 downregulated genes were identified (**Supplementary Figure 1A**). KEGG and GO enrichment analyses of these DEGs revealed significant enrichment in pathways related to disulfidptosis, such as the actin cytoskeleton and cell adhesion functions (**Supplementary Figures 1B, C**). These findings preliminarily suggest a potential link between disulfidptosis and pancreatic cancer (complete enrichment results are provided in **Supplementary Tables 2 and 3**).

### 3.2 Construction of a disulfidptosis-related prognostic model

**Figure 2:**
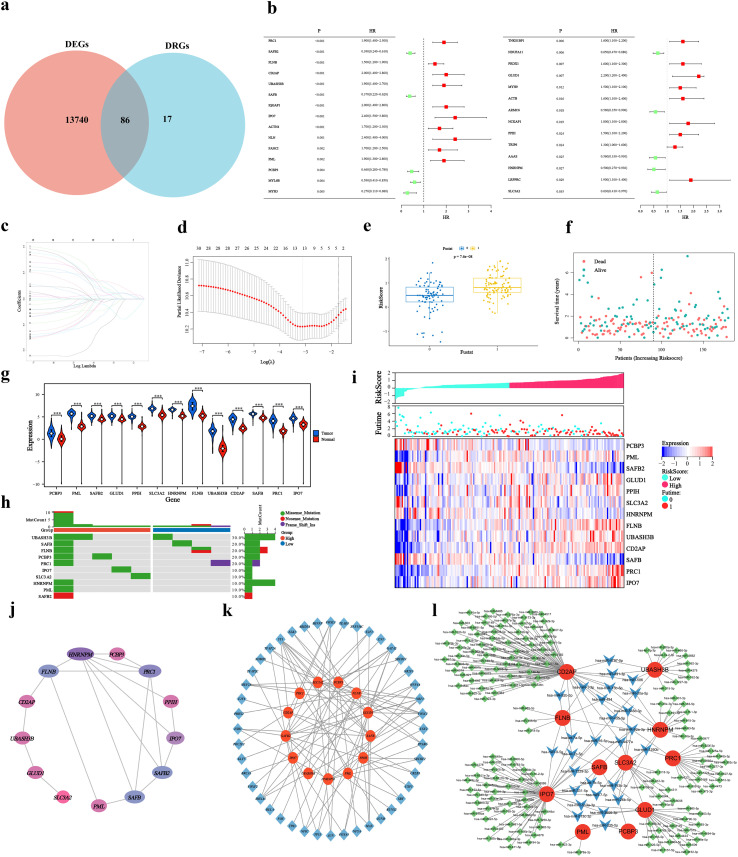
Construction of the disulfidptosis-related prognostic model. **(a)** Venn diagram showing 86 overlapping genes between DRGs and DEGs. **(b)** Forest plot of univariate COX analysis for DRGs (p< 0.05). **(c)** Confidence intervals for each lambda value. **(d)** Trajectory of independent variable coefficients. The optimal lambda is indicated by vertical dashed lines. **(e)** Distribution of risk scores aligned with patient survival status. **(f)** Distribution of survival status and survival time. Green and red dots represent alive and deceased patients, respectively. **(g)** Expression levels of model genes in tumor versus normal tissues. **(h)** Mutation profiles of model genes in high- and low-risk groups. **(i)** Heatmap showing correlations between risk scores and key model gene expression. **(j)** Protein-protein interaction (PPI) network of the model genes. **(k)** Transcription factor regulatory network associated with the model genes. **(l)** miRNA-associated regulatory network of the model genes. *P< 0.05, **P< 0.01, ***P< 0.001, ****P< 0.0001.

From the initial 13,826 highly significant DEGs, an intersection with 103 DRGs yielded 86 pancreatic cancer-associated DRGs (**Figure 2A**). Univariate Cox analysis and LASSO-Cox regression were applied, selecting 13 model genes with non-zero coefficients at the optimal lambda value (**Figures 2B–D**). A disulfidptosis-related prognostic model was subsequently constructed. Patients were stratified into high- and low-risk groups based on the median risk score. The risk score was notably higher in the deceased patient group compared to the surviving group (**Figures 2E, F**). Significant expression differences were observed among the model genes (**Figure 2G**). Mutation analysis indicated that all model genes maintained low mutation frequencies (**Figure 2H**), demonstrating their expression stability and further supporting the reliability of the prognostic model. Additionally, high-risk patients exhibited upregulation of pro-oncogenic genes (UBASH3B, CD2AP, PRC1) and downregulation of protective genes (SAFB, SLC3A2, HNRNPM) (**Figure 2I**). To elucidate the biological coherence of the prognostic signature, we assessed interactions among its constituent genes. Protein-protein interaction network analysis confirmed statistically significant interconnectivity (**Figure 2J**), suggesting functional collaboration. Furthermore, we identified common transcription factors and miRNAs concurrently targeting multiple signature genes (**Figures 2K, L**), indicating coordinated transcriptional and post-transcriptional regulatory programs underlying their co-expression.

### 3.3 Survival prediction and validation of the prognostic model

**Figure 3:**
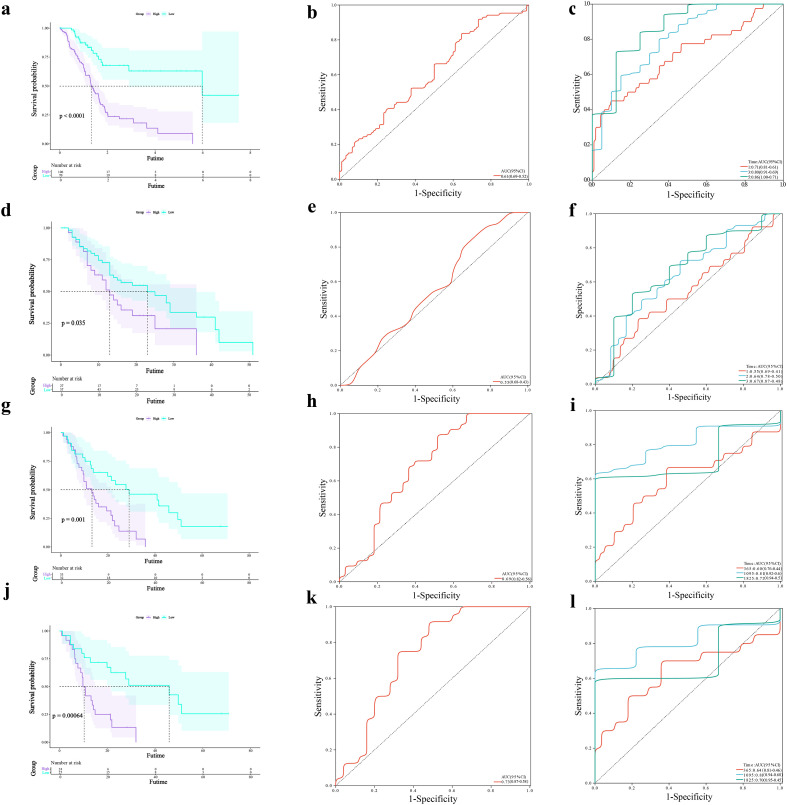
Survival prediction and validation of the prognostic model. **(a)** Kaplan-Meier survival curves for high- and low-risk groups stratified by the DRG-based risk score. **(b)** ROC curve evaluating the sensitivity of the prognostic model. **(c)** Time-dependent ROC curves predicting 1-, 3-, and 5-year overall survival. **(d–f)** KM survival analysis, ROC curves, and time-dependent ROC for the GSE28735 cohort. **(g–i)** Corresponding analyses for the GSE62452 cohort. **(j–l)** Corresponding analyses for the GSE78229 cohort.

Survival analysis revealed that patients in the low-risk group had significantly longer overall survival and a higher median survival time than those in the high-risk group (**Figure 3A**). To evaluate the predictive performance of the prognostic model, the AUC was calculated to be 0.61 (**Figure 3B**). Furthermore, time-dependent ROC analysis showed AUC values of 0.71, 0.80, and 0.86 for 1-, 3-, and 5-year survival, respectively (**Figure 3C**), indicating high predictive accuracy. External validation was performed using three independent datasets (GSE28735, GSE62452, and GSE78229). Consistently, the high-risk group exhibited significantly shorter survival times across all cohorts, and the ROC curve results further confirmed the reliability of the model (**Figures 3D–L**).

### 3.4 Clinical correlation analysis of the DRG prognostic score and development of a diagnostic nomogram

**Figure 4:**
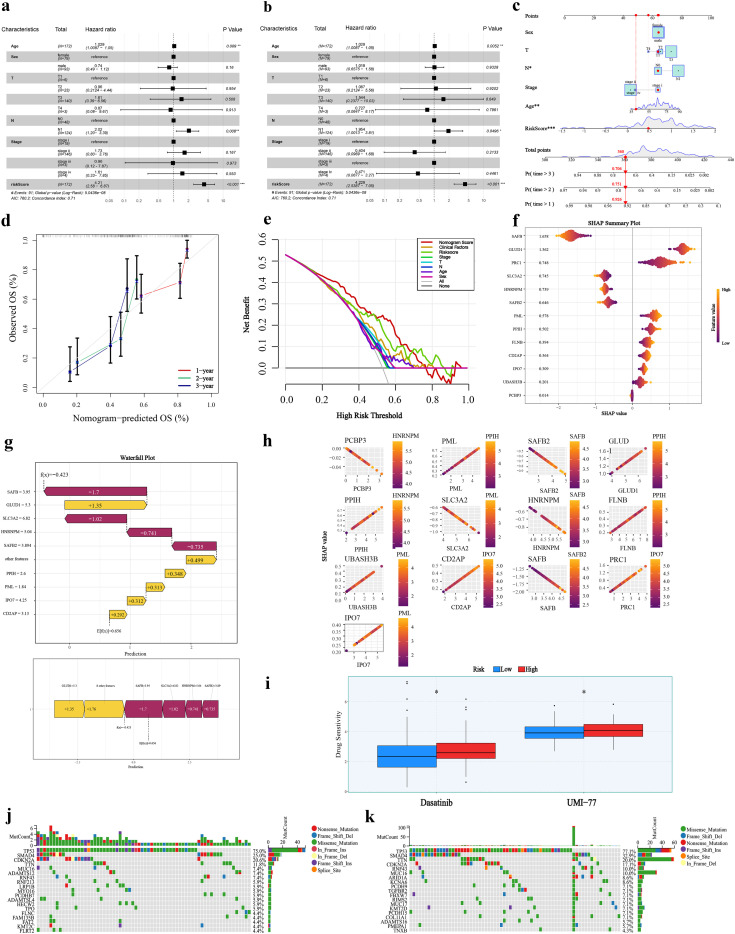
Clinical correlation analysis and diagnostic nomogram construction. **(a, b)** Forest plots from univariate and multivariate COX regression analyses of prognostic risk factors. **(c)** Nomogram integrating clinical features and the risk score to predict 1–3-year survival. **(d)** Calibration curves for 1–3-year survival predictions. **(e)** DCA evaluating the net benefit of the integrated model. **(f)** SHAP summary plot displaying the impact of core genes on model output. **(g)** Waterfall plot illustrating prediction contributions for individual samples. **(h)** Scatter plot relating gene expression levels to SHAP values. **(i)** Comparison of drug sensitivity between risk groups. **(j, k)** Mutational landscapes in high- and low-risk groups.

To further evaluate the independent prognostic value and clinical relevance of the DRGs score, univariate COX regression analysis was first performed. The results indicated that age, N1 stage, and the risk score were significantly associated with patient survival (**Figure 4A**). Multivariate COX regression analysis further confirmed that the risk score serves as an independent prognostic factor for pancreatic cancer patients (**Figure 4B**).

Based on these findings, the risk score was integrated with key clinical characteristics (including age, gender, and T/N stage) to construct a prognostic nomogram for predicting 1–3 year survival probabilities (**Figure 4C**). Calibration curve analysis demonstrated good agreement between the nomogram-predicted outcomes and actual observations (**Figure 4D**). DCA revealed that the integrated nomogram provided a superior net benefit across a wide range of threshold probabilities compared to using clinical variables or the risk score alone (**Figure 4E**), highlighting its potential value for risk stratification and personalized treatment planning in pancreatic cancer patients.

To elucidate the decision-making mechanism of the prognostic model, SHAP analysis was applied to the “univariate COX + Lasso-Cox” disulfidptosis prediction model, quantifying the contribution of each core gene’s transcriptional level to the model’s output. The SHAP summary plot displays the mean SHAP value for each gene, where a larger absolute value indicates a greater impact on the prediction (**Figure 4F**). Waterfall plots visually represent the prediction for individual samples, showing how each feature gene contributes to the final risk score (**Figure 4G**). **Figure 4H** further illustrates the relationship between gene expression levels and their corresponding SHAP values.

Drug sensitivity analysis suggested that patients in the high-risk group may exhibit increased sensitivity to dasatinib (a tyrosine kinase inhibitor) and UMI-77 (an Mcl-1 inhibitor) (**Figure 4I**). Additionally, gene mutation analysis identified TP53 and SMAD4 as the most frequently mutated genes in the cohort (**Figures 4J, K**).

### 3.5 Functional and signaling pathway enrichment analysis of the prognostic model

**Figure 5:**
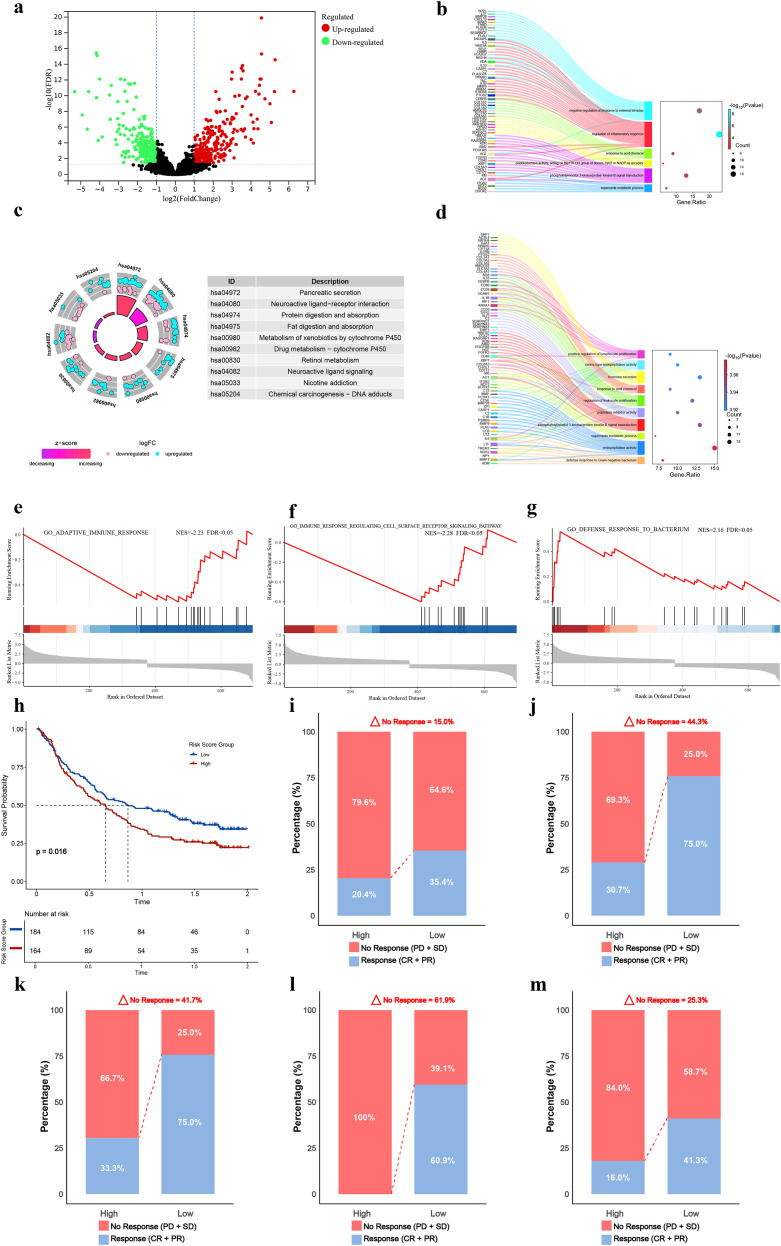
Functional enrichment analysis of the prognostic model. **(a)** Volcano plot of DEGs between risk groups. Red and green dots represent up- and down-regulated genes (|log_2_FC| > 1, adjusted p< 0.05). **(b)** GO terms associated with disulfidptosis-related mechanisms. **(c, d)** Top 10 enriched KEGG and GO pathways. **(e–g)** GSEA plots of immune-related pathways enriched in DEGs from the high-risk group. **(H, I)** Survival difference between risk groups **(h)** and response rate difference **(i)** in the IMvigor210 cohort. **(j–m)** Response rate differences between risk groups in the GSE163839, GSE100797, GSE78220, and GSE165252 cohorts.

Differential gene expression analysis between the high- and low-risk groups identified 393 upregulated and 350 downregulated genes (**Figure 5A**). GO functional and KEGG pathway enrichment analyses revealed significant associations with redox-related pathways (**Figures 5B–D**; complete enrichment results are provided in **Supplementary Tables 4 and 5**). Further GSEA analysis indicated that differentially expressed genes in the high-risk group were significantly enriched in five immune-related pathways, including immune response regulation and adaptive immune response (NES > 1, p< 0.05) (**Figures 5E–G**). The remaining GSEA results are shown in **Supplementary Figure 2**. These findings suggest that disulfidptosis-related signatures are associated with redox homeostasis and immune function, highlighting their potential relevance to patient prognosis in pancreatic cancer. Given the functional differences in tumor-associated immune states between the high- and low-risk groups and to provide more direct biological evidence supporting the clinical utility of our model for treatment guidance, we analyzed immunotherapy response in the IMvigor210 cohort (**Figures 5H, I**) and multiple GEO datasets (GSE100797, GSE165252, GSE78220, GSE163839) (**Figures 5J–M**). The results consistently showed that patients in the high-risk group had a significantly higher proportion of non-responders to immunotherapy compared to those in the low-risk group.

### 3.6 Immune characterization of the prognostic model

**Figure 6:**
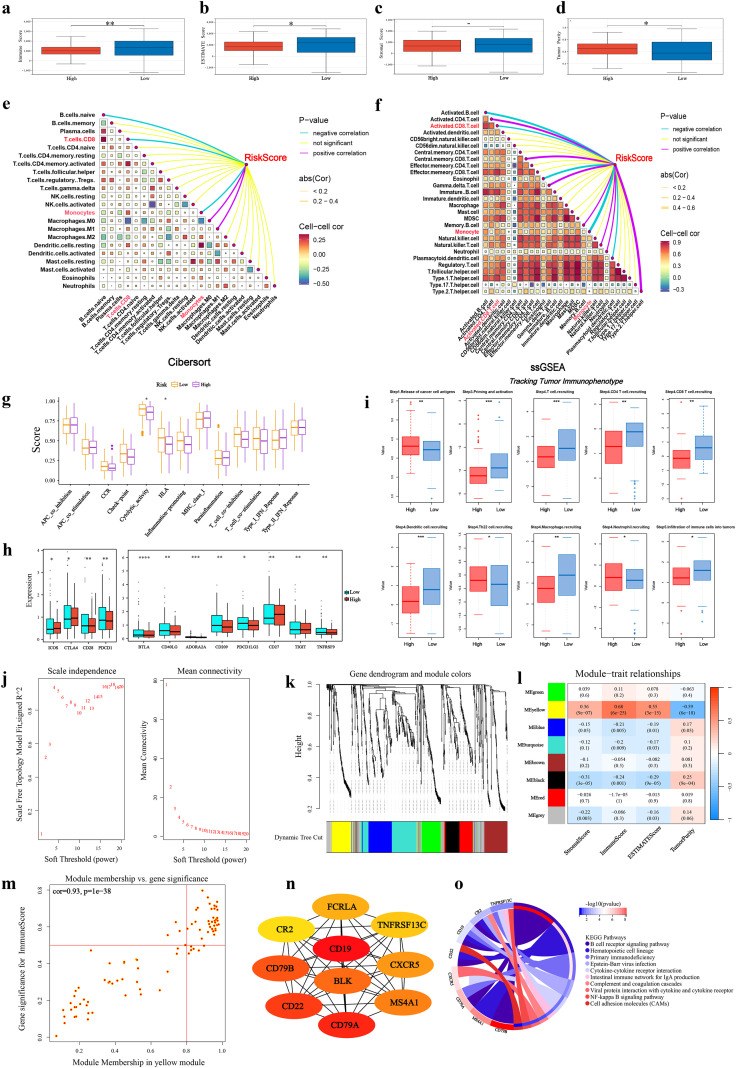
Immune characterization based on the prognostic model. **(a–d)** Violin plots comparing stromal, immune, ESTIMATE scores, and tumor purity between risk groups. **(e, f)** Correlation between risk scores and immune cell infiltration levels assessed by CIBERSORT and ssGSEA. **(g)** Differences in immune-related functions between risk groups via ssGSEA. **(h)** Differential expression of immune checkpoints. **(i)** Differences in TIP analysis results between high- and low-risk groups. **(j)** Scale-free fit index and mean connectivity for soft-thresholding power selection. Red line indicates R² = 0.9 at β = 4. **(k)** Gene dendrogram and module colors from WGCNA. **(l)** Heatmap of module-trait correlations between gene modules and immune scores. **(m)** Scatter plot of gene significance for immune score versus module membership in the yellow module. **(n)** Protein-protein interaction network of hub genes. **(o)** KEGG enrichment analysis of immune-related genes. *P< 0.05, **P< 0.01, ***P< 0.001, ****P< 0.0001.

Analysis using the ESTIMATE algorithm showed no significant difference in stromal scores, whereas immune scores and tumor purity differed significantly between the risk groups (**Figures 6A–D**). The CIBERSORT and ssGSEA algorithms were applied to evaluate immune cell infiltration levels and their correlation with the risk score (**Figures 6E, F**). CIBERSORT results indicated a negative correlation between the high-risk group and CD8^+^ T cell infiltration (**Figure 6E**, **S3**, **Supplementary Table 6**). ssGSEA revealed significant differences in the abundance of 11 immune cell types, with various T cell subtypes showing prominent variation (**Figure 6F**, **S3**, **Supplementary Table 7**). Functional analysis demonstrated significant differences between risk groups in cytokine activity, HLA-related pathways (**Figure 6G**), four classical immune checkpoints (ICOS, CD28, CTLA4, PDCD1), and eight emerging immune checkpoints (BTLA, CD40LG, ADORA2A, CD209, CD27, PDCD1LG2, TIGIT, TNFRSF9) (**Figure 6H**). Further validation using the Tracking Tumor Immunophenotype analysis confirmed that the high-risk group exhibited significantly impaired recruitment of multiple T-cell subsets and diminished overall immune cell infiltration compared to the low-risk group (**Figure 6I**).

To further investigate immune-related differentially expressed genes, WGCNA was performed using ESTIMATE scores as traits. At a soft-thresholding power of β = 4, the network met scale-free topology criteria (scale-free R² = 0.9) with a low mean connectivity (**Figure 6J**). Eight distinct modules were identified (**Figure 6K**). Module–trait correlation analysis showed that the yellow module had the highest correlation with immune scores (**Figure 6L**), which was further validated by module membership analysis (**Figure 6M**; additional correlations are shown in **Supplementary Figures 4A–C**). Functional analysis of genes in the yellow module indicated their involvement in various tumor immune functions and a negative correlation with tumor purity (**Figures 6N, O**, **Supplementary Figure 4D**). These genes also showed strong correlations with multiple immune cell types from ssGSEA, such as CD4^+^ T cells, CD8^+^ T cells, and memory CD8^+^ T cells (**Supplementary Figure 4E**), further supporting distinct immune landscapes between the high- and low-risk groups.

### 3.7 Identification of the prognostic biomarker UBASH3B and functional characterization

**Figure 7:**
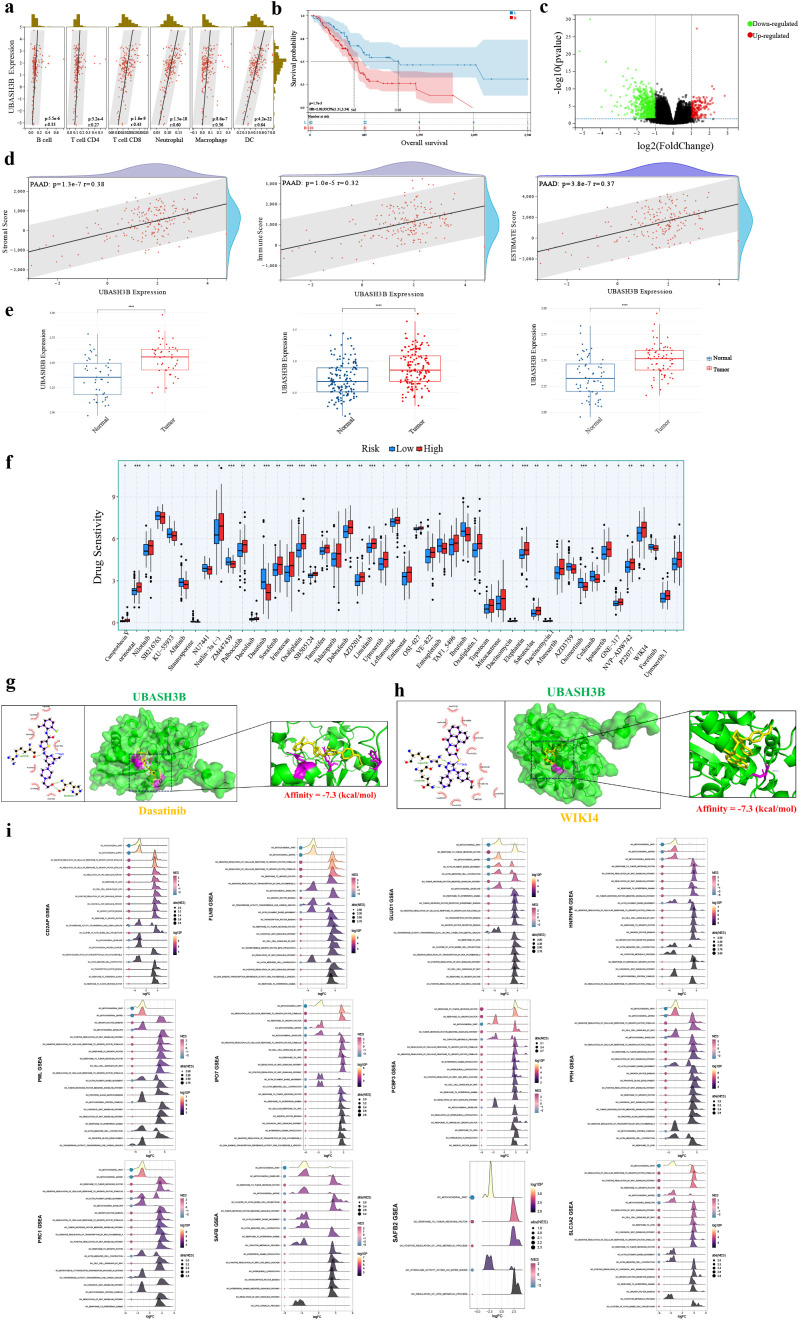
Identification and functional validation of UBASH3B as a prognostic biomarker. **(a)** Correlation between UBASH3B expression and immune cell infiltration scores from TIMER. **(b)** Survival analysis between high and low UBASH3B expression groups. **(c)** Volcano plot of DEGs between UBASH3B expression groups. **(d)** Correlation between UBASH3B expression and ESTIMATE scores. **(e)** External validation of UBASH3B expression in GEO datasets. **(f)** Differential drug sensitivity between UBASH3B expression groups. **(g, h)** Docking results of UBASH3B with Dasatinib **(g)** and WIKI4 **(h)**. **(i)** GSEA enrichment analysis results for the other 12 model genes.

To further investigate the impact of the prognostic model on immune function and survival outcomes in pancreatic cancer patients, immune cell infiltration analysis was performed for the 13 model genes. Results demonstrated that UBASH3B showed the highest correlation with six types of immune cells (**Figure 7A**, **S5**). Additionally, UBASH3B significantly influenced patient survival (**Figure 7B**, **S6**). UBASH3B was therefore identified as a potential prognostic biomarker for pancreatic cancer. Subsequent validation using RF and SVM-RFE algorithms confirmed the stability and accuracy of UBASH3B as a prognostic biomarker (**Supplementary Figures 7A–C**).

Differential gene expression analysis between high and low UBASH3B expression groups (**Figure 7C**) and subsequent KEGG enrichment analysis revealed associations with pancreatic cancer immune responses, including T/B cell differentiation and activation, cytokine activity, and innate and adaptive immune responses (**Supplementary Figures 7D–H**). Correlation analysis between UBASH3B expression and ESTIMATE scores further supported its association with immune infiltration (**Figure 7D**). UBASH3B also showed differential expression across multiple cancer types (**Supplementary Figure 7I**).

External validation using the GSE28735, GSE62452, and GSE71229 datasets confirmed the differential expression pattern of UBASH3B (**Figure 7E**). Drug sensitivity analysis comparing high and low UBASH3B expression groups identified 48 agents with significant differences in sensitivity (**Figure 7F**), including anti-pancreatic cancer drugs such as dasatinib, irinotecan (a DNA topoisomerase inhibitor), dabrafenib (a BRAF inhibitor), and entinostat (an HDAC inhibitor). Using the CIDE database, we further validated the concurrent upregulation of UBASH3B at both the protein and transcriptional levels in pancreatic cancer tissues (**Supplementary Figures 7J, K**). Analysis of UBASH3B expression across immune cell subtypes revealed its predominant enrichment in immunosuppressive cells, such as Tregs (**Supplementary Figure 7L**). To identify high-confidence potential therapeutic agents, we performed drug sensitivity analysis in the GSE71729 and GSE62452 datasets to narrow down candidate compounds. This process consistently identified Dasatinib and WIKI4 as the two drugs showing highly significant differences in sensitivity (all p< 0.01) (**Supplementary Figures 7M, N**). Subsequent molecular docking studies confirmed high binding affinity between UBASH3B and both drugs (**Figures 7G, H**). Additionally, evaluation of the IMvigor210 cohort (**Supplementary Figure 7O**) and multiple GEO immunotherapy datasets (**Supplementary Figures 7P–R**) also demonstrated that patients with high UBASH3B expression exhibited a significantly higher proportion of non-responders to immunotherapy compared to those with low expression. Functional enrichment of the remaining model genes beyond UBASH3B revealed their convergent roles in key pathways, including tumor immune response, Wnt/growth factor signaling, tumorigenesis, and disulfidptosis-related metabolism (**Figure 7I**), highlighting their potential collective relevance to pancreatic cancer progression through associations with disulfidptosis, immune modulation, and oncogenic signaling.

### 3.8 Single-cell RNA sequencing data analysis

**Figure 8:**
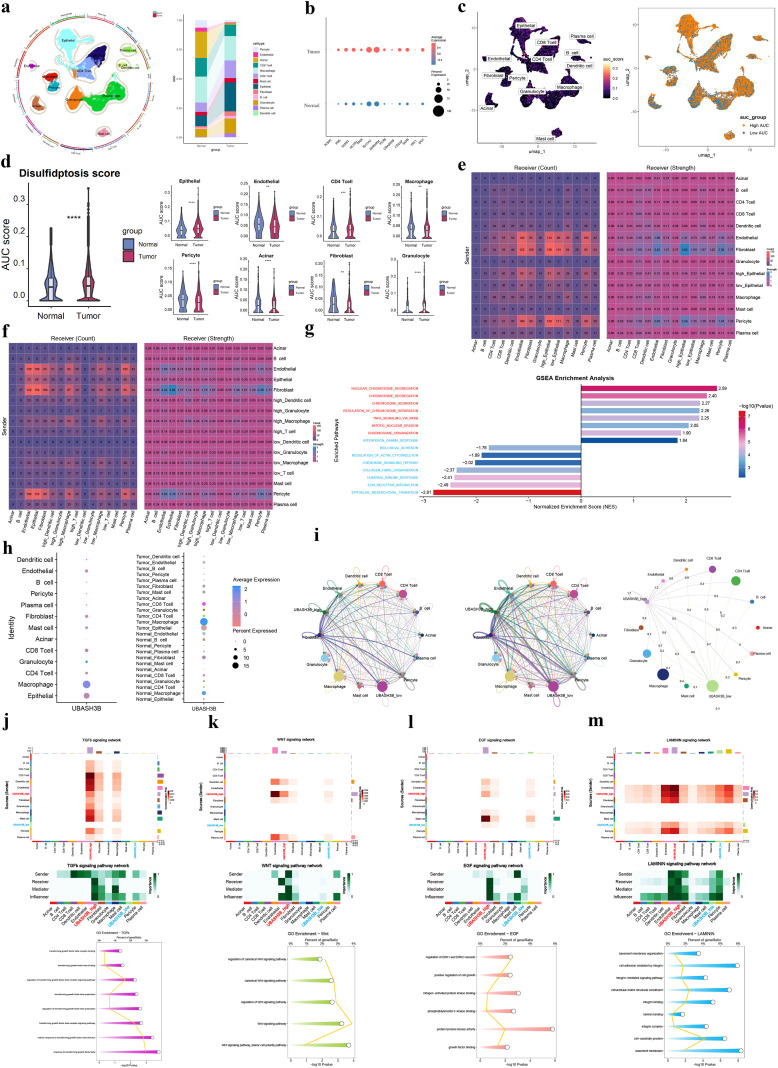
Single-cell profiling of disulfidptosis-related features. **(a)** Cell type annotation and composition in tumor and normal tissues. **(b)** Expression of model genes in tumor versus normal tissues. **(c)** Distribution of disulfidptosis scores across cell types in pancreatic cancer. **(d)** Overall and cell-type-specific disulfidptosis scores in tumor and normal cells. **(e, f)** Heatmaps of interaction strength and number in tumor and immune cell communication. **(g)** GSEA of DEGs between high and low disulfidptosis score groups. **(h)** UBASH3B expression across cell types and groups. **(i)** CellChat analysis of interaction count and strength, stratified by UBASH3B expression. **(j–m)** Communication probability and functional roles of TGF-β, Wnt, EGF, and LAMININ pathways.

Single-cell RNA sequencing data (GSE155698) underwent quality control, Harmony integration for batch effect correction (**Supplementary Figures 8A, B**), and cell clustering analysis, resulting in the identification of 20 distinct clusters (**Supplementary Figure 8C**). These clusters were annotated into 13 major cell types, including epithelial cells and endothelial cells (**Figure 8A**, **S8D**). Comparison of prognostic model gene expression between tumor and normal tissues revealed that all model genes except PCBP3 were expressed at higher levels in tumor samples (**Figure 8B**). The heterogeneity of the disulfidptosis score, calculated based on model genes, was further examined across different cell types in tumor and normal tissues (**Figure 8D**). Results demonstrated that disulfidptosis scores were significantly elevated in tumor tissues and in specific tumor cell populations compared to their normal counterparts (**Figures 8C, D**). To determine the predominant cellular origin of disulfidptosis within the pancreatic tumor microenvironment, we examined the expression patterns of the core disulfidptosis regulators, SLC7A11 and NCKAP1, across distinct cell subsets. Both genes showed substantially higher expression levels and frequencies in tumor epithelial cells compared to other cell populations (**Supplementary Figure 8E**). Consistently, comparative analysis of disulfidptosis scores across cell subsets further supported that tumor cells represent the primary site of disulfidptosis in pancreatic cancer (**Supplementary Figure 8F**). **Figure 8E and 8F** illustrate that tumor epithelial cells and immune cells (such as T cells) with high disulfidptosis scores exhibited active cell-cell communication.

GSEA of differentially expressed genes in tumor epithelial cells between high and low disulfidptosis score groups revealed significant activation of cell division and proliferation-related pathways, including “Chromosome segregation” and “Mitotic nuclear division”, while pathways such as “ECM receptor interaction”, “Epithelial mesenchymal transition”, and “Interferon-γ response” were significantly suppressed (**Figure 8G**). Additionally, T cell activation and cytokine-related functions were associated with the disulfidptosis score (**Supplementary Figure 8G**).

The key gene UBASH3B was found to be significantly upregulated in tumor tissues, particularly in epithelial cells and macrophages (**Figure 8H**). Cell communication analysis indicated that UBASH3B-high tumor cells exhibited increased interaction numbers and communication strength with other cells within the tumor microenvironment (**Figure 8I**). Further analysis identified that UBASH3B-high tumor epithelial cells showed enhanced communication strength in four key pathways: Wnt, TGF-β, LAMININ, and EGF (**Figures 8J–M**), particularly the TGF-β and Wnt signaling pathways. This enhanced communication likely influences tumor cell-extracellular matrix interactions (**Supplementary Figure 8H**) and inhibits the function of tumor-killing cells such as T cells and NK cells (**Supplementary Figure 8I**). These findings were further validated by GSEA (**Supplementary Figure 8J**). To further elucidate the cell communication network involving UBASH3B within the pancreatic cancer microenvironment, we performed ligand-receptor interaction analysis. The results revealed that UBASH3B-high cells function as key recipients of multiple signaling inputs, whereas fibroblasts constitute the primary source of signal output (**Supplementary Figure 9A**). In the TGF-β signaling axis, UBASH3B-high cells predominantly act as signaling inputs, with signals mainly originating from immune cell subsets such as T cells and macrophages (**Supplementary Figure 9B**). In contrast, within the Wnt signaling axis, UBASH3B-high cells primarily serve as signal senders, with endothelial cells acting as the main signal recipients (**Supplementary Figure 9C**). Subsequent ligand-receptor specificity analysis identified TGF-β1 as the key ligand in UBASH3B-associated TGF-β communication, primarily interacting with the TGF-βR2 and TGF-βR1 receptor complex, with ACVR1 also potentially involved (**Supplementary Figures 9D–G**). For Wnt signaling, Wnt10a and Wnt7b were identified as the predominant ligand subtypes, interacting with LRP6 and FZD6 or FZD4 (**Supplementary Figures 9H–L**).

### 3.9 The role of UBASH3B and disulfidptosis in the origin of tumor cells

**Figure 9:**
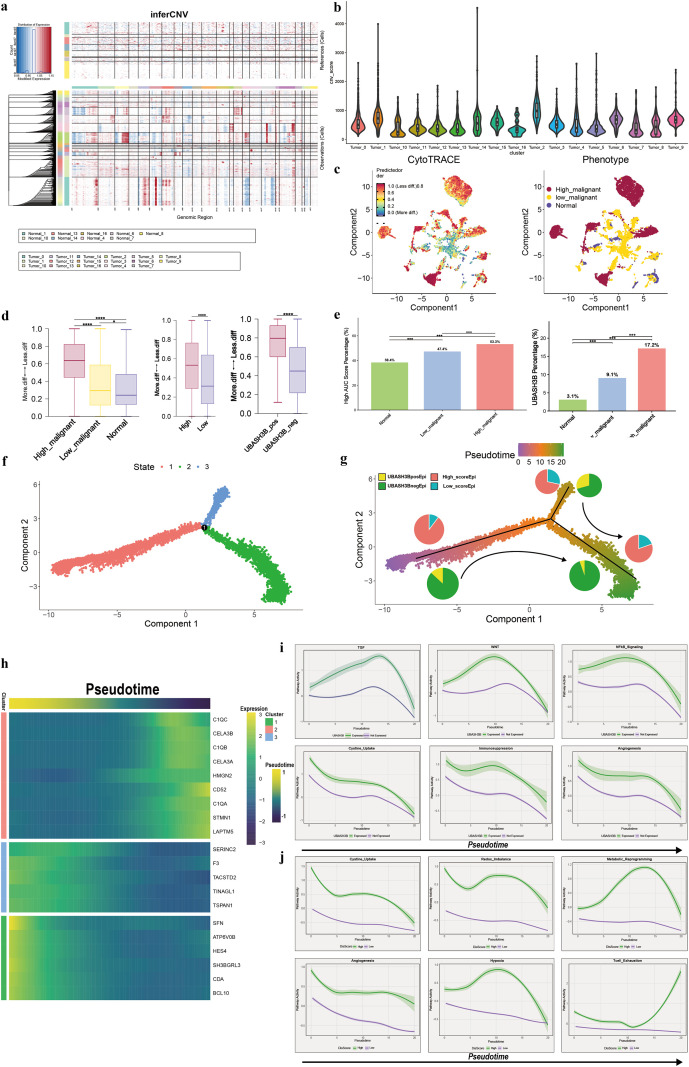
Reverse pseudotime analysis reveals the association of UBASH3B and disulfidptosis with the origin of pancreatic cancer epithelial cells. **(a)** Heatmap of inferCNV across epithelial cells. **(b)** The violin plot of CNV score. **(c)** UMAP of epithelial cells colored by CytoTRACE-predicted stemness and malignancy grade. **(d)** CytoTRACE scores across subgroups defined by malignancy, UBASH3B expression, and disulfidptosis score. **(e)** Proportion of high-malignancy cells stratified by UBASH3B expression **(right)** and disulfidptosis score (left). **(f)** Pseudotime trajectory of epithelial cells reconstructed using Monocle2, colored by inferred developmental state. **(g)** Reverse pseudotime analysis reveals changes of the percentage of UBASH3B expression and disulfidptosis scores. **(h)** Heatmap of DEGs across distinct branches of the developmental trajectory. **(i, j)** Comparative pseudotime analysis of tumor activity related pathways in different subgroup of **(i)** UBASH3B expression and **(j)** disulfidptosis scores. *P< 0.05, **P< 0.01, ***P< 0.001, ****P< 0.0001.

To further investigate the characteristics of disulfidptosis and UBASH3B in pancreatic tumorigenesis, epithelial cells were extracted for subsequent analysis. Initially, inferCNV was employed to identify tumor cells with varying degrees of malignancy (**Figures 9A, B**). Subsequent CytoTRACE analysis delineated the stemness dynamics within the epithelial compartment (**Figure 9C**). The CytoTRACE results indicated a progressive increase in stemness (corresponding to a loss of differentiation) concomitant with rising malignant potential. Notably, this low-differentiation phenotype was also prominently observed in cells exhibiting high UBASH3B expression and elevated disulfidptosis scores (**Figure 9D**). Furthermore, cells with high UBASH3B expression or high disulfidptosis scores (calculated based on model genes) were found to be enriched along the developmental trajectory toward highly malignant tumor cell states (**Figure 9E**).

To investigate the potential roles of UBASH3B and disulfidptosis in the cellular origins of pancreatic cancer, we performed pseudotime trajectory analysis on epithelial cells using Monocle (**Figure 9F**). The inferred developmental trajectory revealed a consistent decline in the proportions of both UBASH3B-expressing cells and cells with high disulfidptosis scores as pseudotime progressed, reaching their lowest levels by the terminal developmental stage (**Figure 9G**). Differential expression analysis across distinct inferred developmental branches identified early-stage signatures characterized by genes such as CELA3A/B, HMGN2, and STMN1, whereas later stages were marked by significant upregulation of genes including TINAGL1, ATP6V08, CDA (**Figure 9H**).Reverse pseudotime analysis of key pathways—encompassing tumor progression, tumor immunity, and disulfidptosis-related metabolism—stratified by UBASH3B expression or disulfidptosis score, revealed a consistent decreasing trend along the trajectory for several pathways. These included the TGF-β pathway, Wnt signaling, NF-κB signaling, cystine uptake, redox balance, and tumor immunosuppression. Importantly, cells with high UBASH3B expression and high disulfidptosis score exhibit stronger characteristics throughout the entire reverse pseudotime.

### 3.10 Spatial transcriptomic data analysis

**Figure 10:**
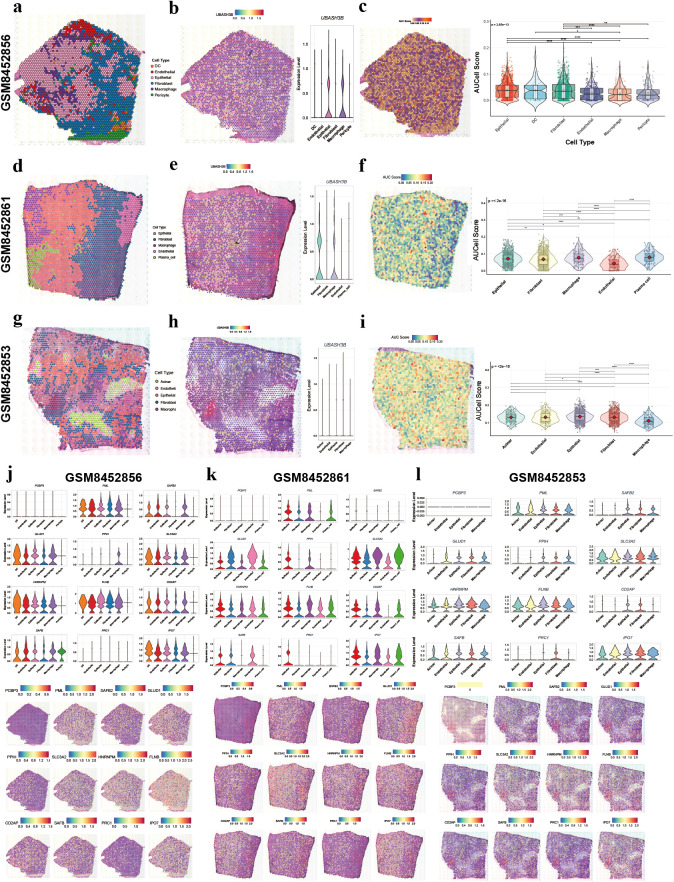
Spatial transcriptomic profiling of disulfidptosis activity and core gene expression in three independent PDAC samples. (**a-c**) Analysis of an initial PDAC sample. **(a)** Spatial distribution map of annotated major cell types. **(b)** Violin plot comparing UBASH3B expression levels across cell types **(right)** and spatial expression map of UBASH3B (left). **(c)** Spatial map of the disulfidptosis activity score calculated by AUCell **(right)** and violin plot comparing the disulfidptosis score across cell types (left). **(d–f)** and **(g–i)** show the identical analytical workflow and consistent results for two independent validation PDAC samples. **(j–l)** Violin plots and spatial expression maps for the other 12 prognostic model genes across the three respective samples.

Cells were annotated into six major types, including epithelial cells and fibroblasts, whose spatial distribution patterns are visualized in **Figure 10A**. Spatial mapping of the key gene UBASH3B revealed that tumor epithelial cells with high UBASH3B expression were predominantly localized adjacent to fibroblasts (**Figure 10B**). Given that fibroblasts represent a major source of Wnt ligands and TGF-β in the tumor microenvironment, this spatial proximity may have implications for intercellular signaling, but further studies are needed. UBASH3B expression was also significantly higher in epithelial cells than in other cell types (**Figure 10B**).

Similar to the single-cell RNA sequencing analysis, disulfidptosis scores were calculated for cells in the spatial transcriptomic data (**Figure 10C**). Epithelial cells displayed one of the highest disulfidptosis scores among all cell populations (**Figure 10C**), consistent with the scoring patterns observed in the single-cell analysis. This identical analytical workflow was applied to two additional, independent PDAC samples for validation. The results were highly consistent across all three cohorts, confirming that epithelial cells consistently exhibit the highest UBASH3B expression and disulfidptosis activity (**Figures 10D–I**). Finally, the spatial expression patterns of the other 12 prognostic model genes were examined across the three samples. The majority of these genes also showed elevated expression in epithelial cells, reinforcing their association with tumor cells (**Figures 10J–L**).

### 3.11 Silencing UBASH3B suppresses pancreatic cancer proliferation and metastasis

**Figure 11:**
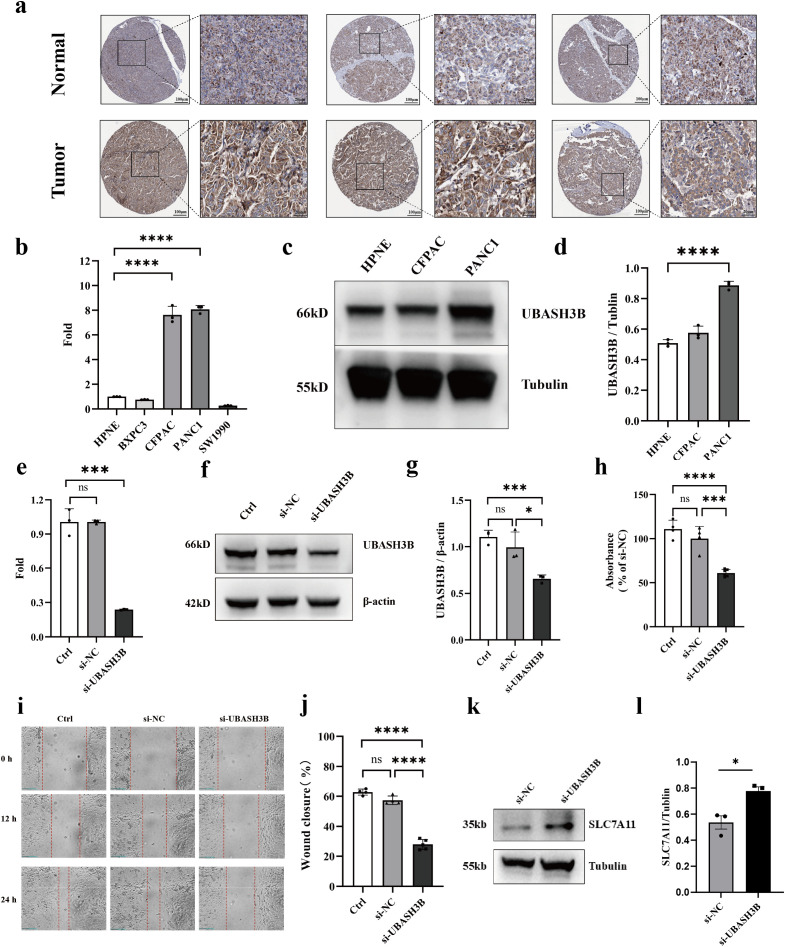
Silencing UBASH3B suppresses proliferation and metastasis in pancreatic cancer. **(a)** IHC staining of UBASH3B in tumor and normal tissues. **(b)** UBASH3B mRNA levels were measured by RT-qPCR in the normal pancreatic epithelial cell line HPNE and various pancreatic cancer cell lines. Data were analyzed using the 2^(-ΔΔCT) method. **(c)** UBASH3B protein levels were assessed by Western blotting. **(d)** Western blot images were quantified using ImageJ software. Relative protein expression is presented as the ratio of the target protein gray value to that of Tubulin. **(e)** RT-qPCR validation of UBASH3B expression in PANC1 cells following siRNA-mediated knockdown. **(f, g)** UBASH3B protein levels in PANC1 cells after siRNA transfection were evaluated by Western blotting **(f)** and quantified using ImageJ **(g)**. Relative protein expression levels are shown as the ratio of UBASH3B gray value to that of β-actin. **(h)** The effect of UBASH3B knockdown on the proliferation of PANC1 cells was measured by the CCK-8 assay. **(i)** Representative images (0h, 12h, 24h) from the wound healing assay, demonstrating the effect of UBASH3B knockdown on the migration of PANC1 cells. **(J)** Quantitative analysis of the wound healing assay. The wound closure rate at 24 hours was calculated using ImageJ as the percentage of the healed area relative to the initial wound area at 0 hours. *P< 0.05, **P< 0.01, ***P< 0.001, ****P< 0.0001.

Immunohistochemistry results from the HPA database demonstrated stronger staining intensity for UBASH3B in pancreatic cancer tissues compared to normal tissues, indicating upregulated UBASH3B protein expression in tumors (**Figure 11A**). RT-qPCR analysis identified higher UBASH3B transcript levels in CFPAC and PANC1 cell lines (**Figure 10B**). Western blotting further confirmed significantly elevated UBASH3B protein expression in PANC1 cells (**Figures 11C, D**). Consequently, PANC1 was selected for subsequent functional studies. UBASH3B expression was effectively silenced in PANC1 cells using siRNA, as validated by both RT-qPCR and Western blotting (**Figures 11E–G**).

**Figure 12:**
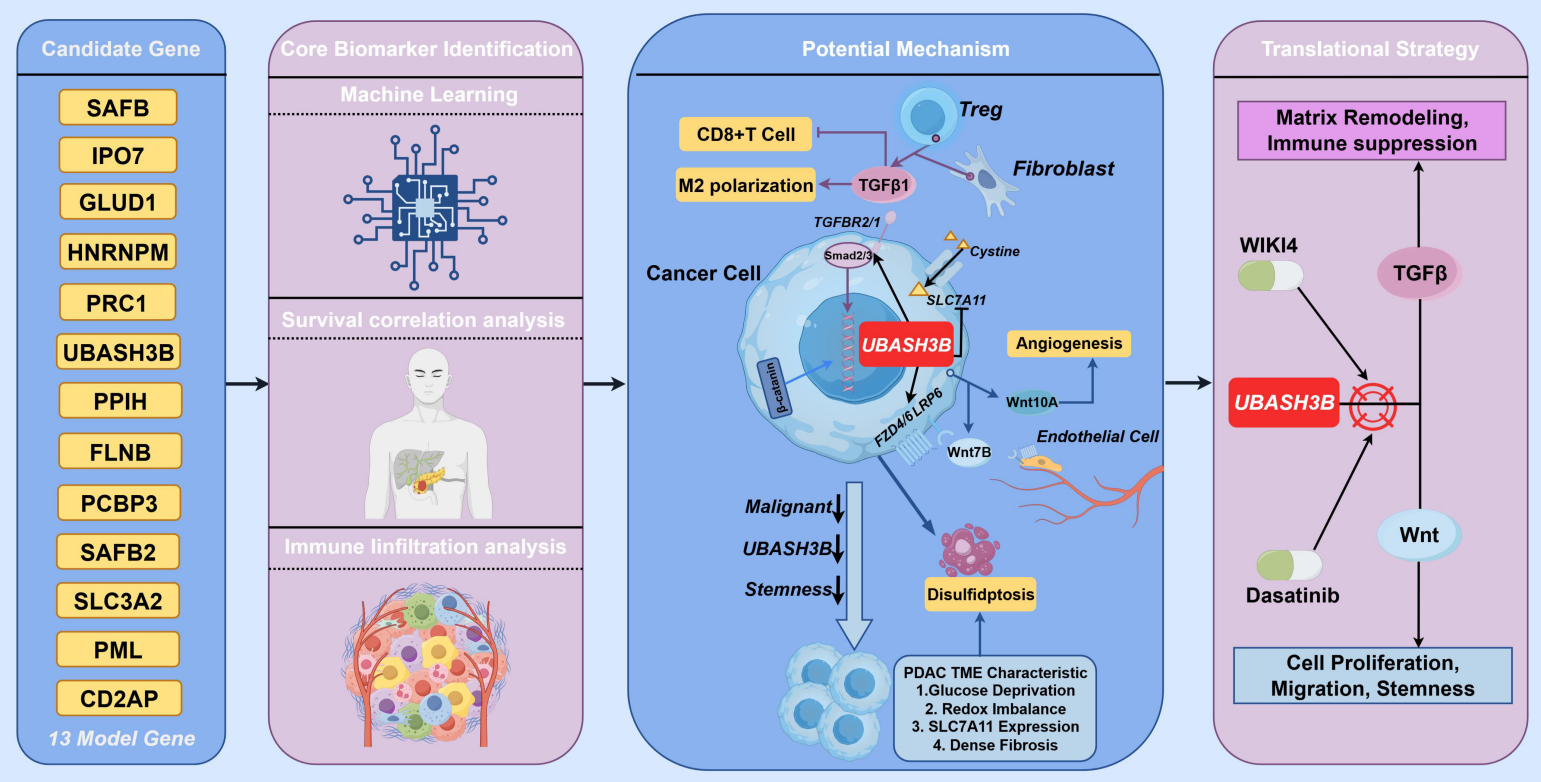
Potential regulatory mechanisms and transformation strategies of UABSH3B as a core biomarker.

CCK-8 assays revealed that UBASH3B knockdown significantly inhibited tumor cell proliferation (**Figure 11H**). Migration assays also indicated that reduced UBASH3B expression effectively suppressed tumor cell migration (**Figures 11I, J**). Given that SLC7A11 is a key mediator of disulfidptosis, we analyzed changes in its expression following UBASH3B knockdown. We found that SLC7A11 expression was upregulated upon UBASH3B silencing (**Figures 11K, L**), suggesting that UBASH3B knockdown may potentially promote the induction of disulfidptosis. This observation is also in alignment with the aforementioned finding that UBASH3B inhibition suppresses pancreatic cancer cell proliferation. These findings collectively suggest that high expression of UBASH3B may play a potential role in promoting tumor cell proliferation and migration, thereby contributing to disease progression and poor prognosis in pancreatic cancer patients. Taken together, the potential multi-layered regulatory role of UBASH3B within the pancreatic cancer tumor microenvironment is illustrated in **Figure 12**.

## 4 Discussion

The incidence and mortality rates of pancreatic cancer continue to rise, posing significant challenges for diagnosis and treatment due to its insidious onset, rapid progression, and high malignancy. RCD pathways in tumor cells, including ferroptosis, autophagy, and more recently, disulfidptosis, have become a major focus in cancer research. These pathways not only induce tumor cell death but also remodel the tumor immune microenvironment and influence immunotherapy efficacy (38). However, the role of disulfidptosis in remodeling the cancer microenvironment remains in its early exploratory stages. Research in this area holds significant potential for discovering novel therapeutic approaches and improving the efficacy of conventional treatments. PDAC represents a compelling research target in this context due to its unique TME. The characteristic features of the PDAC microenvironment, marked by redox imbalance and severe glucose deprivation, constitute the metabolic conditions inducing disulfidptosis. Furthermore, key molecular drivers of disulfidptosis, such as SLC7A11, are frequently overexpressed in PDAC (12, 16). This intrinsic connection not only underscores a close link between disulfidptosis and pancreatic cancer progression but also opens a novel perspective for metabolic intervention, offering a potential therapeutic avenue to address its clinical challenges, including late diagnosis and poor response to existing therapies.

In this study, we conducted a preliminary investigation to explore the potential associations of disulfidptosis with metabolic vulnerabilities and microenvironmental features in PDAC. A prognostic model incorporating 13 key genes (PCBP3, PML, SAFB2, GLUD1, PPIH, SLC3A2, HNRNPM, FLNB, UBASH3B, CD2AP, SAFB, PRC1, IPO7) was developed through univariate and LASSO-Cox regression analyses. This model robustly stratifies patient outcomes. Multi-omics analyses revealed intricate interactions among redox homeostasis, cytoskeletal dynamics, and immune evasion. By integrating machine learning algorithms with diverse biological features, we also identified UBASH3B as a core prognostic biomarker. The model demonstrated superior predictive performance compared to traditional staging systems and was validated across multiple independent cohorts. Multivariate Cox regression further confirmed the risk score as an independent prognostic factor. Furthermore, the model provides guidance for personalized therapy, identifying differential drug sensitivity to UMI-77 and dasatinib between risk subgroups. Prior evidence suggests dasatinib inhibits pancreatic cancer metastasis, while UMI-77 effectively blocks tumor cell growth, indicating their promising therapeutic potential (39, 40).

Functional enrichment analysis revealed significant differences between the high- and low-risk groups in cell adhesion junctions, the actin cytoskeleton network, and cellular redox balance. These findings align with the established core mechanism of disulfidptosis, wherein glucose starvation or oxidative stress in SLC7A11-high cells depletes NADPH, leading to aberrant intracellular disulfide bond accumulation and subsequent cytoskeletal collapse. Furthermore, comprehensive evaluation using multiple algorithms demonstrated that the high-risk group exhibits an immune-desert phenotype characterized by reduced infiltration of CD8^+^T cells, accompanied by upregulation of multiple immune checkpoint molecules. Consistent with this, GSEA revealed significant differences between high- and low-risk groups in pathways such as immune response regulation and adaptive immune response, suggesting an implied association between disulfidptosis activity and immunosuppressive microenvironments. To gain higher-resolution insights, we performed single-cell and spatial transcriptomic analyses. Analysis revealed that the core disulfidptosis regulators, SLC7A11 and NCKAP1, are predominantly expressed in epithelial cells, where the disulfidptosis score is markedly higher than in any immune cell population. Furthermore, tumor epithelial cells with elevated disulfidptosis scores exhibited active proliferation-related pathways and enhanced communication with neighboring cells through key immunomodulatory pathways. These findings suggest that dysregulation of disulfidptosis in pancreatic cancer is not only associated with cell death but also correlates with features of the immunosuppressive tumor microenvironment. Given that disulfidptosis occurs primarily in tumor cells rather than immune cells, this association may originate from tumor cell-intrinsic processes, underscoring the need for further functional investigations.

Under normal conditions, disulfidptosis can initiate anti-tumor immunity through multiple mechanisms. The release of classic DAMPs, including ATP and HMGB1, triggers immunogenic cell death. Additionally, metabolic collapse in dying tumor cells may alleviate nutrient competition with surrounding immune effectors, thereby restoring their functional capacity (41). These immunostimulatory properties have been validated across multiple studies, underscoring the therapeutic potential of inducing disulfidptosis to enhance antitumor immunity (42, 43). However, emerging evidence suggests that disulfidptosis may also engage counterregulatory pathways that promote immune tolerance. For instance, tumor cell SLC7A11-mediated cystine uptake causing T cells nutrient deprivation, disrupting glutathione synthesis and redox homeostasis, thereby driving T cell exhaustion and dysfunction (41). Simultaneously, the cystine/glutamate exchange activity of SLC7A11 drives extracellular glutamate accumulation during disulfidptosis, thereby promoting Treg proliferation and suppressive function via metabotropic glutamate receptor signaling (44, 45). Deletion of SLC7A11 in pancreatic tumor cells results in reduced Treg accumulation and alleviated systemic inflammation (46). In addition, disulfidptosis may lead to the efflux of oxidative metabolites such as ROS and oxidized glutathione, potentially driving immune cells toward a tolerogenic phenotype (47). Thus, whether disulfidptosis promotes immunity or tolerance is linked to the balance of opposing signals, and clarifying its context-dependent immunological effects is a key focus of current research. Future efforts should focus on identifying the molecular determinants that govern the switch between immunosuppression and immunogenicity.

Through an integrative analysis combining survival analysis, immune infiltration assessment, and multiple machine learning algorithms, we identified UBASH3B as a core gene within the model, significantly impacting overall survival and immune cell infiltration, and thus proposed as a potential prognostic biomarker for PDAC. While UBASH3B has been reported to promote proliferation, invasion, and metastasis in malignancies like breast and prostate cancer (48–50), its role in pancreatic cancer remained unclear. Our multi-omics analysis reveals that the high expression of UBASH3B in a specific subset of tumor epithelial cells, coupled with enhanced intercellular communication via TGF-β and Wnt pathways, suggests its potential role for UBASH3B in the aggressive phenotype. Based on these findings, we propose that UBASH3B represents a candidate for further investigation as a therapeutic target in PDAC. In-silico drug sensitivity screening and molecular docking analyses identified WIKI4 and dasatinib as candidate compounds with strong predicted binding affinity for UBASH3B, both of which have been reported to exert therapeutic effects against pancreatic cancer progression, suggesting that further exploration of UBASH3B-targeted inhibitors is warranted (39, 51). While these computational findings are encouraging, further experimental validation will be essential to determine whether targeting UBASH3B could achieve therapeutic objectives.

Although our study implicates UBASH3B and disulfidptosis in pancreatic cancer, several limitations should be acknowledged. The prognostic model relies on retrospective public data, where heterogeneous sample collection and limited outcome details may introduce selection bias and affect generalizability. Prospective multi-center validation with standardized protocols is needed. While we have established the role of UBASH3B in promoting tumor cell proliferation and migration, the precise signaling pathways through which it regulates disulfidptosis and its specific role in remodeling the tumor microenvironment require deeper mechanistic investigation. In particular, the direct experimental evidence to causally link tumor cell disulfidptosis to impaired immune cell function is lacking. Future studies employing co-culture systems and cell-type-specific genetic models are essential to functionally validate this proposed immunosuppressive mechanism. Furthermore, our analysis is primarily based on transcriptomic data. Integrating multi-omics approaches, such as metabolomics to trace redox metabolites, proteomics to quantify cytoskeletal changes, and epigenomics to profile promoter methylation level of disulfidptosis core genes, would provide a more comprehensive understanding of disulfidptosis in pancreatic cancer. Future studies utilizing both *in vitro* and *in vivo* models targeting UBASH3B will be essential for functionally elucidating this proposed immunosuppressive mechanism. Finally, translating this model into a clinical tool faces practical hurdles, including developing a robust, cost-effective detection method and defining clear decision thresholds for integration into diagnostic pathways. Addressing these gaps will strengthen the mechanistic foundation and clinical potential of our findings.

In summary, this study established and validated a DRGs prognostic model for pancreatic cancer. The analysis revealed a potential link between disulfidptosis activity and an immunosuppressive microenvironment, identifying UBASH3B as a key prognostic marker. These findings contribute to our understanding of the interplay among metabolism, immunity, and tumor evolution in pancreatic cancer, and offer insights that may inform development of novel prognostic biomarkers and combination therapeutic strategies.

## 5 Conclusion

Through bioinformatic analysis, key prognostic genes were identified from DRGs, leading to the development and validation of a novel prognostic prediction model. This model enhances the understanding of how disulfidptosis-related signatures correlate with clinical outcomes, offering a basis for further exploration of individualized therapeutic approaches. Based on the model, patients were stratified into high- and low-risk subgroups, and distinct immune characteristics and functional differences were delineated between these subgroups. Furthermore, UBASH3B was proposed as a novel biomarker for predicting prognosis in pancreatic cancer patients.

## Data Availability

The original contributions presented in the study are included in the article/**Supplementary Material**. Further inquiries can be directed to the corresponding author.

## Supplementary material

Supplementary Figure 1 **(A)** Volcano plot of differentially expressed genes between tumor and normal tissues. Thresholds were set at |log_2_ Fold Change| > 2 and adjusted p-value< 0.05, with downregulated and upregulated genes colored in green and red, respectively. **(B)** GO enrichment bubble plot of DEGs, highlighting disulfidptosis-related pathways. **(C)** KEGG enrichment bubble plot of DEGs, showing pathways associated with disulfidptosis.

Supplementary Figure 2 **(A, B)** KEGG pathway enrichment analyses related to high- and low-risk groups.

Supplementary Figure 3 **(A)** Immune cell types with significantly different proportions estimated by the CIBERSORT algorithm. **(B)** Immune cell types showing significant differences in abundance based on ssGSEA.

Supplementary Figure 4 Scatter plots of gene significance versus module membership in the yellow module for stromal score **(A)**, ESTIMATE score **(B)**, and tumor purity **(C, D)** Heatmap of correlations between immune-related genes and the ESTIMATE score. **(E)** Heatmap of correlations between immune-related genes and immune cell abundance.

Supplementary Figure 5 Immune infiltration analysis for **(A)** SAFB, **(B)** CD2AP, **(C)** FLNB, **(D)** GLUD1, **(E)** HNRNPM, **(F)** PRC1, **(G)** PPIH, **(H)** PCBP3, **(I)** PML, **(J)** SAFB2, **(K)** SLC3A2, and **(L)** IPO7.

Supplementary Figure 6 Survival analysis for **(A)** SAFB, **(B)** CD2AP, **(C)** FLNB, **(D)** GLUD1, **(E)** HNRNPM, **(F)** PRC1, **(G)** PPIH, **(H)** PCBP3, **(I)** PML, **(J)** SAFB2, **(K)** SLC3A2, and **(L)** IPO7.

Supplementary Figure 7 **(A)** Random Forest model: relationship between the number of trees and error rate. **(B)** Genes ranked by relative importance. **(C)** Feature selection using the SVM-RFE algorithm. **(D-H)** GSEA of differentially expressed genes between high and low expression groups, showing disulfidptosis-related pathways. **(I)** UBASH3B expression across multiple tumor types and normal tissues. **(J, K)** Transcriptional **(J)** and protein-level **(K)** expression differences of UBASH3B in the CPTAC database. **(L)** Expression profile of UBASH3B across different immune cell types. **(M, N)** Drug sensitivity analysis results from GSE71729 **(M)** and GSE62452 **(N)**. **(O, R)** Differences in treatment response rates between UBASH3B high- and low-expression groups in the IMvigor210 **(O)**, GSE78220 **(P)**, GSE78220 **(Q)**, and GSE165252 **(R)** cohorts.

Supplementary Figure 8 **(A, B)** Dimensionality reduction distribution of single cells before and after batch effect removal. **(C)** All cells grouped into 20 clusters. **(D)** Expression of marker genes for each cluster. **(E)** Bubble plots of SLC7A11 and NCKAP1 expression. **(F)** Comparison of disulfidptosis scores across cell subsets. **(G)** GO enrichment analysis of DEGs between high and low disulfidptosis score groups. **(H, I)** KEGG functional enrichment analyses related to disulfidptosis **(H)** and tumorigenesis/immune pathways **(I)**. **(J)** GSEA results.

Supplementary Figure 9 (A) The importance of the role of individual cell subtypes in cellular communication. **(B, C)** The importance of the role of individual cell subtypes in TGF-β signal pathway **(B)** and Wnt signal pathway **(C)**. **(D)** TGF-β signaling-related ligand–receptor interaction probability bubble plot between UBASH3B and various cell subpopulations. **(E–G)** Cross-talk of TGF-β signaling-related key ligand–receptor pairs across distinct cell populations. **(H)** Wnt signaling-related ligand–receptor interaction probability bubble plot between UBASH3B and various cell subpopulations. **(I-L)** Cross-talk of Wnt signaling-related key ligand–receptor pairs across distinct cell populations.
