# Supplementary figures and images for: The role of disulfidptosis-driven tumor microenvironment remodeling in pancreatic cancer progression
Source: Front Immunol. 2026 Mar 10;17:1747560. doi: 10.3389/fimmu.2026.1747560 (PMC13008902; doi:10.3389/fimmu.2026.1747560)

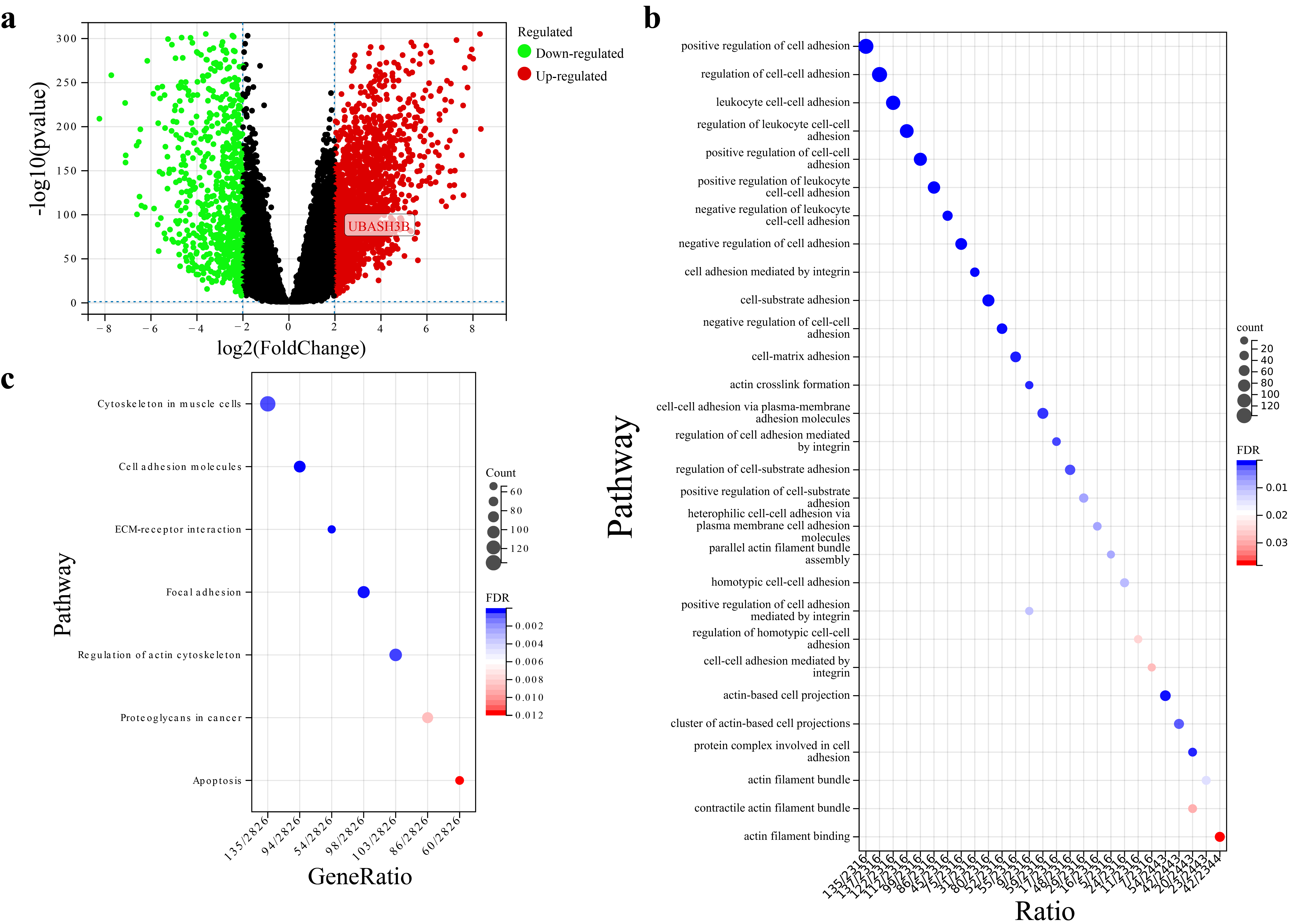

Supplement: Supplementary Figure 1 — (A) Volcano plot of differentially expressed genes between tumor and normal tissues. Thresholds were set at |log2 Fold Change| > 2 and adjusted p-value< 0.05, with downregulated and upregulated genes colored in green and red, respectively. (B) GO enrichment bubble plot of DEGs, highlighting disulfidptosis-related pathways. (C) KEGG enrichment bubble plot of DEGs, showing pathways associated with disulfidptosis. [file Image1.tif]

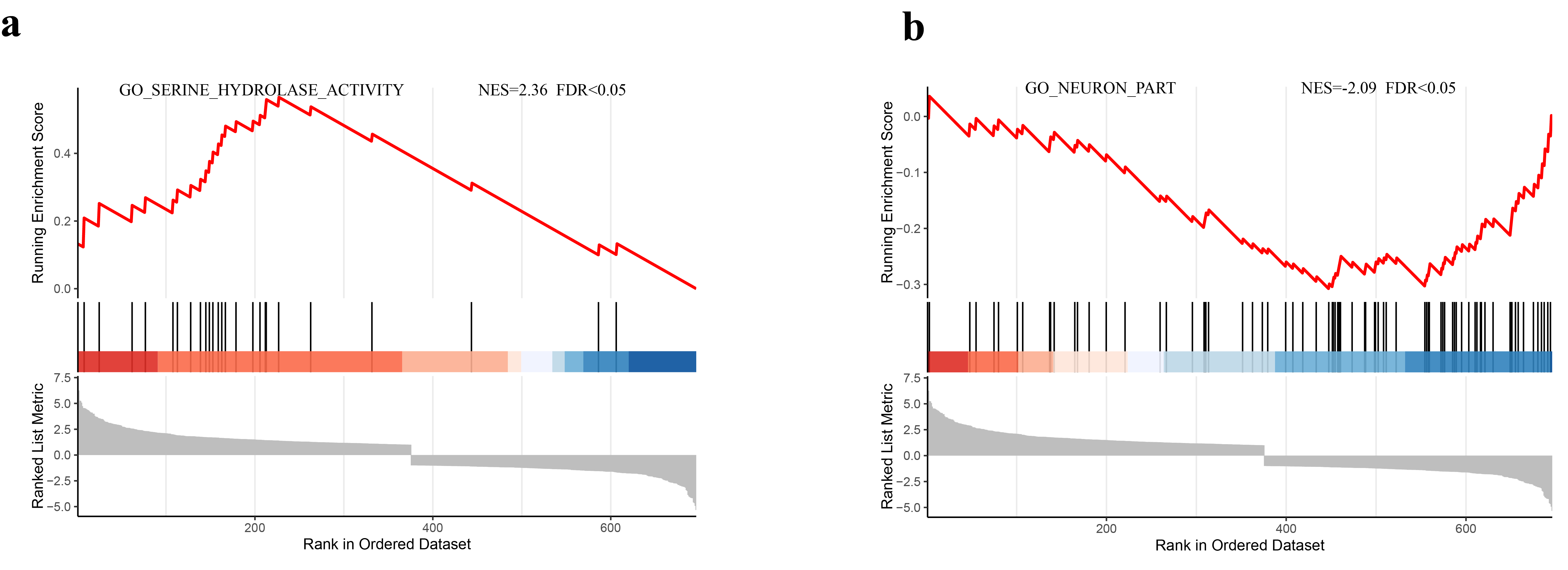

Supplement: Supplementary Figure 2 — (A, B) KEGG pathway enrichment analyses related to high- and low-risk groups. [file Image2.tif]

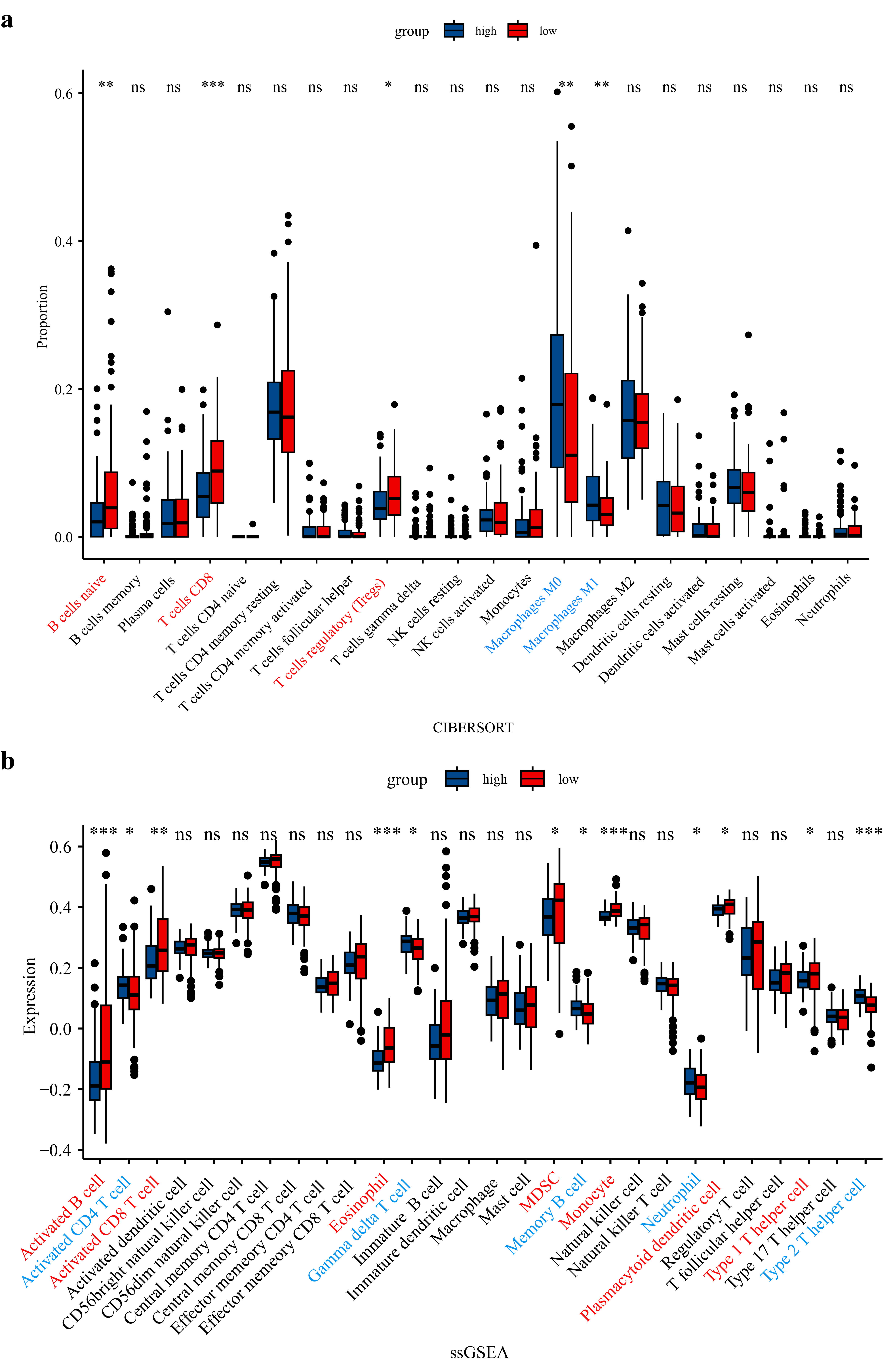

Supplement: Supplementary Figure 3 — (A) Immune cell types with significantly different proportions estimated by the CIBERSORT algorithm. (B) Immune cell types showing significant differences in abundance based on ssGSEA. [file Image3.tif]

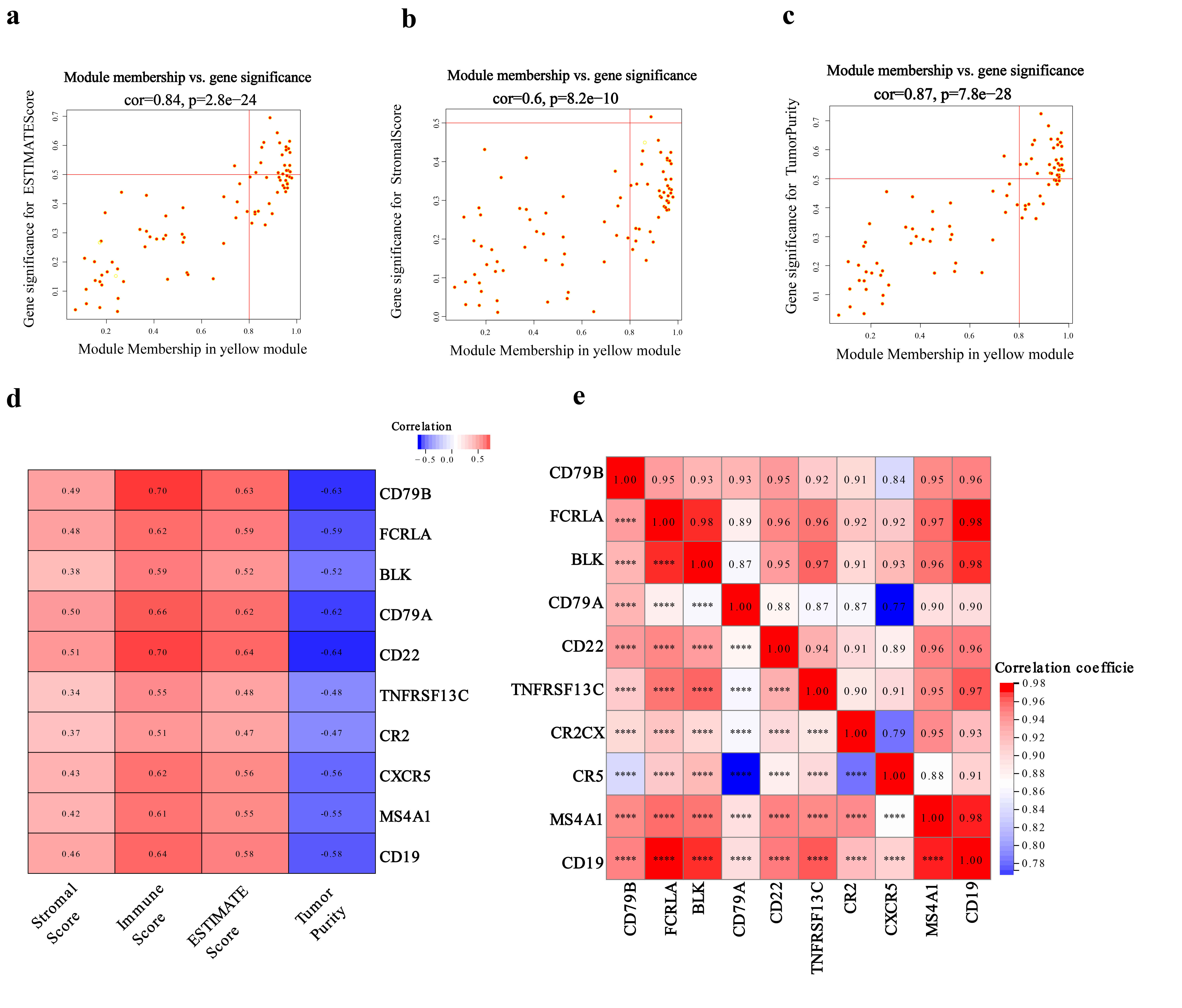

Supplement: Supplementary Figure 4 — Scatter plots of gene significance versus module membership in the yellow module for stromal score (A), ESTIMATE score (B), and tumor purity (C, D) Heatmap of correlations between immune-related genes and the ESTIMATE score. (E) Heatmap of correlations between immune-related genes and immune cell abundance. [file Image4.tif]

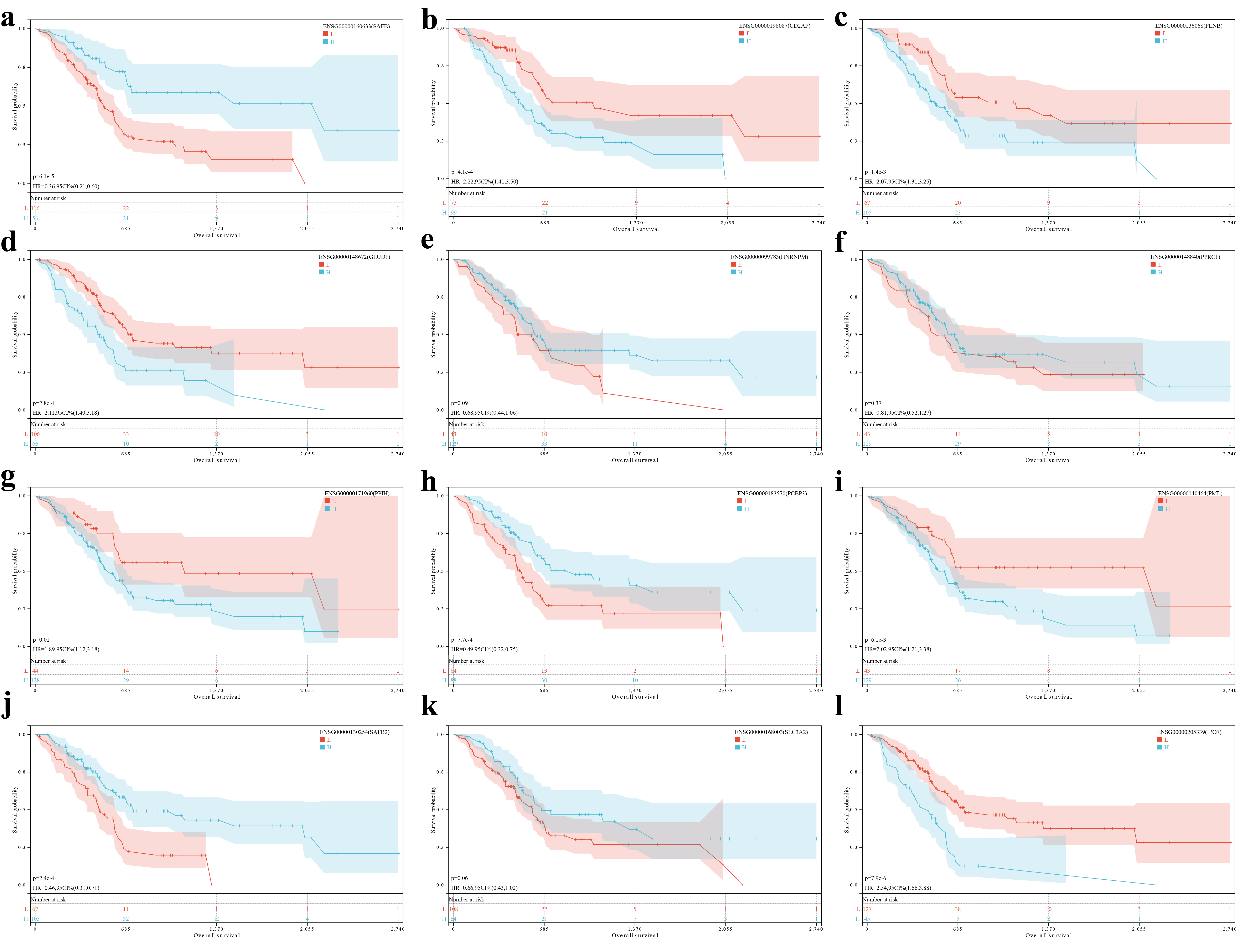

Supplement: Supplementary Figure 5 — Immune infiltration analysis for (A) SAFB, (B) CD2AP, (C) FLNB, (D) GLUD1, (E) HNRNPM, (F) PRC1, (G) PPIH, (H) PCBP3, (I) PML, (J) SAFB2, (K) SLC3A2, and (L) IPO7. [file Image5.tif]

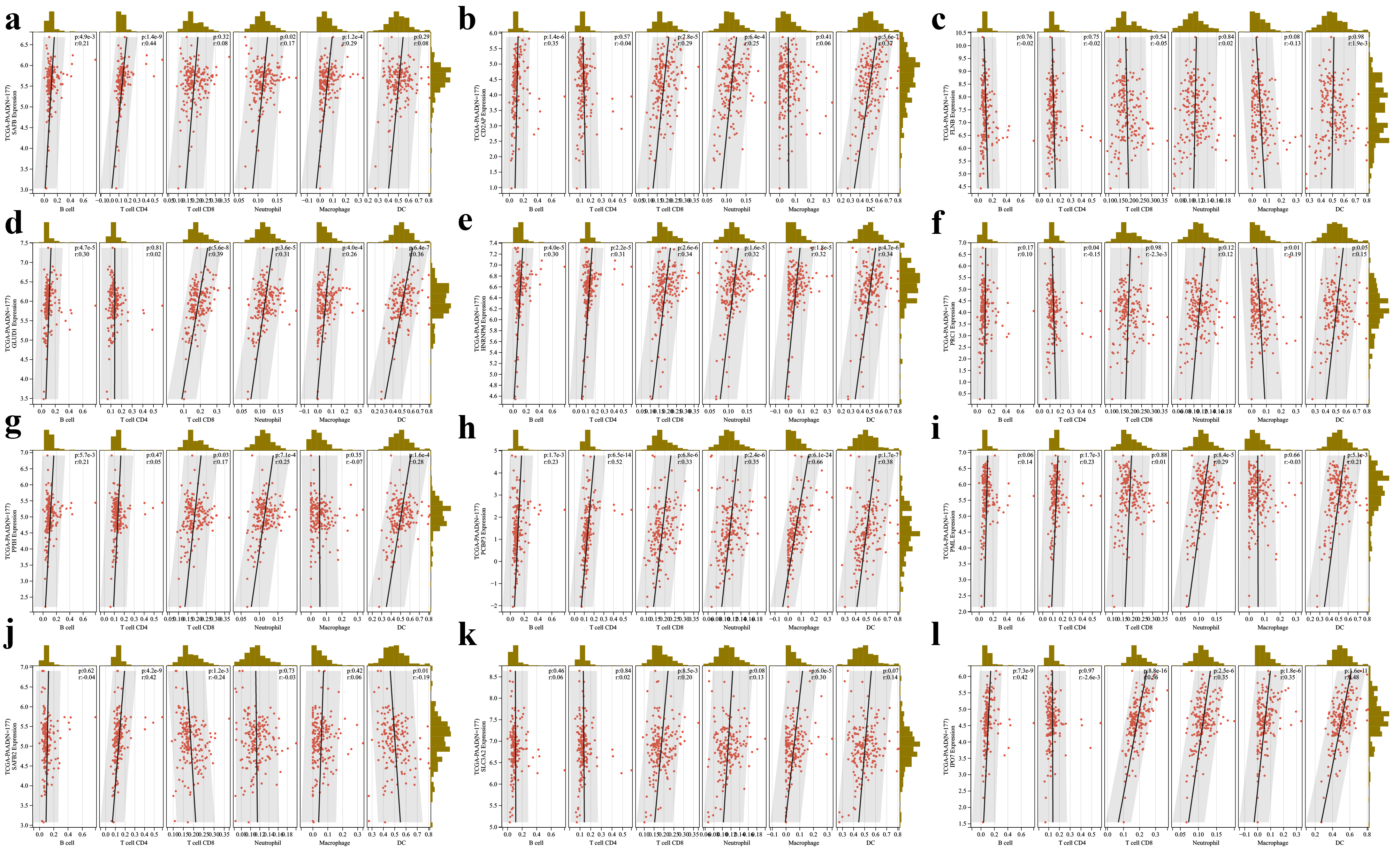

Supplement: Supplementary Figure 6 — Survival analysis for (A) SAFB, (B) CD2AP, (C) FLNB, (D) GLUD1, (E) HNRNPM, (F) PRC1, (G) PPIH, (H) PCBP3, (I) PML, (J) SAFB2, (K) SLC3A2, and (L) IPO7. [file Image6.tif]

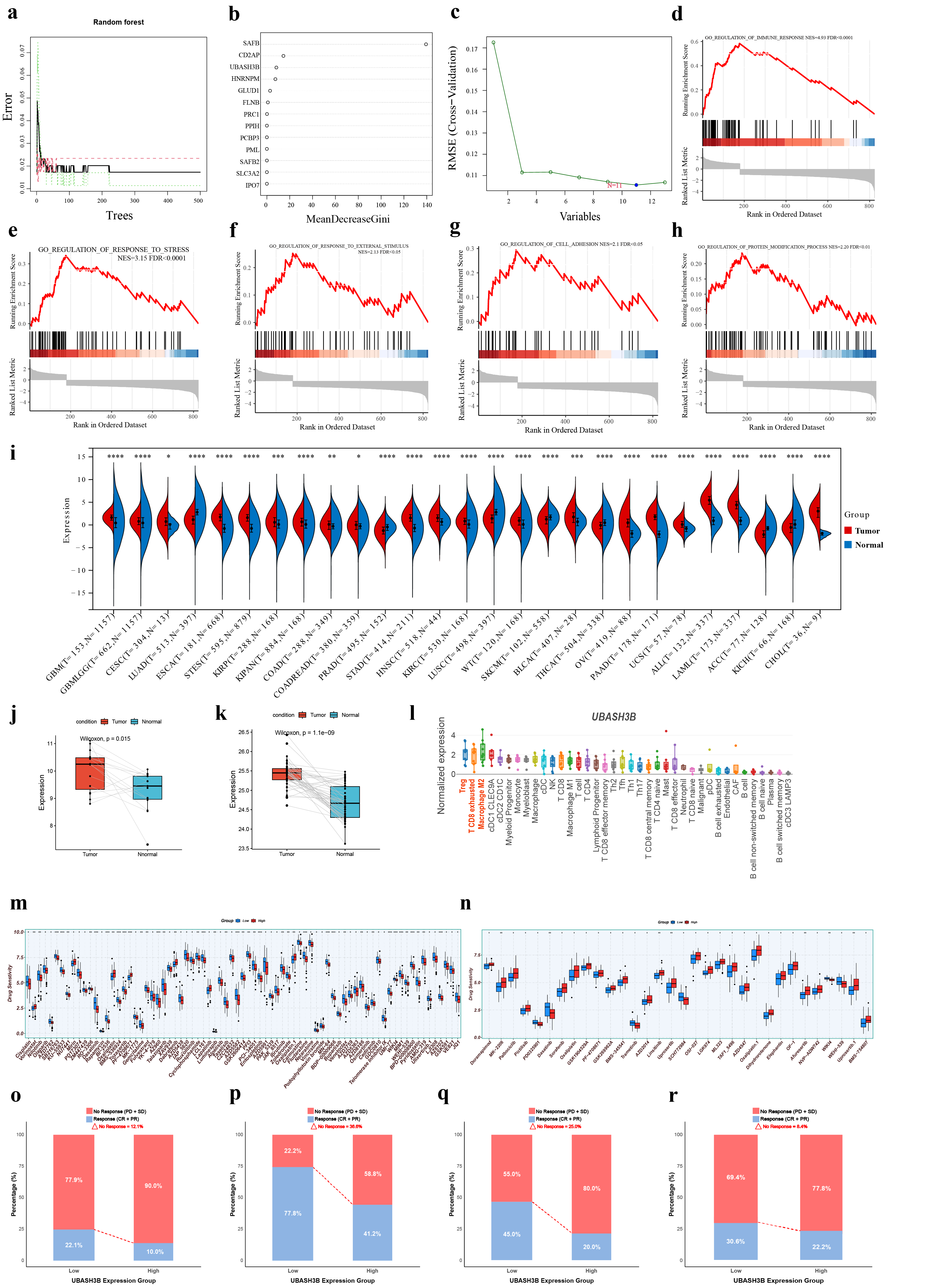

Supplement: Supplementary Figure 7 — (A) Random Forest model: relationship between the number of trees and error rate. (B) Genes ranked by relative importance. (C) Feature selection using the SVM-RFE algorithm. (D-H) GSEA of differentially expressed genes between high and low expression groups, showing disulfidptosis-related pathways. (I) UBASH3B expression across multiple tumor types and normal tissues. (J, K) Transcriptional (J) and protein-level (K) expression differences of UBASH3B in the CPTAC database. (L) Expression profile of UBASH3B across different immune cell types. (M, N) Drug sensitivity analysis results from GSE71729 (M) and GSE62452 (N). (O, R) Differences in treatment response rates between UBASH3B high- and low-expression groups in the IMvigor210 (O), GSE78220 (P), GSE78220 (Q), and GSE165252 (R) cohorts. [file Image7.tif]

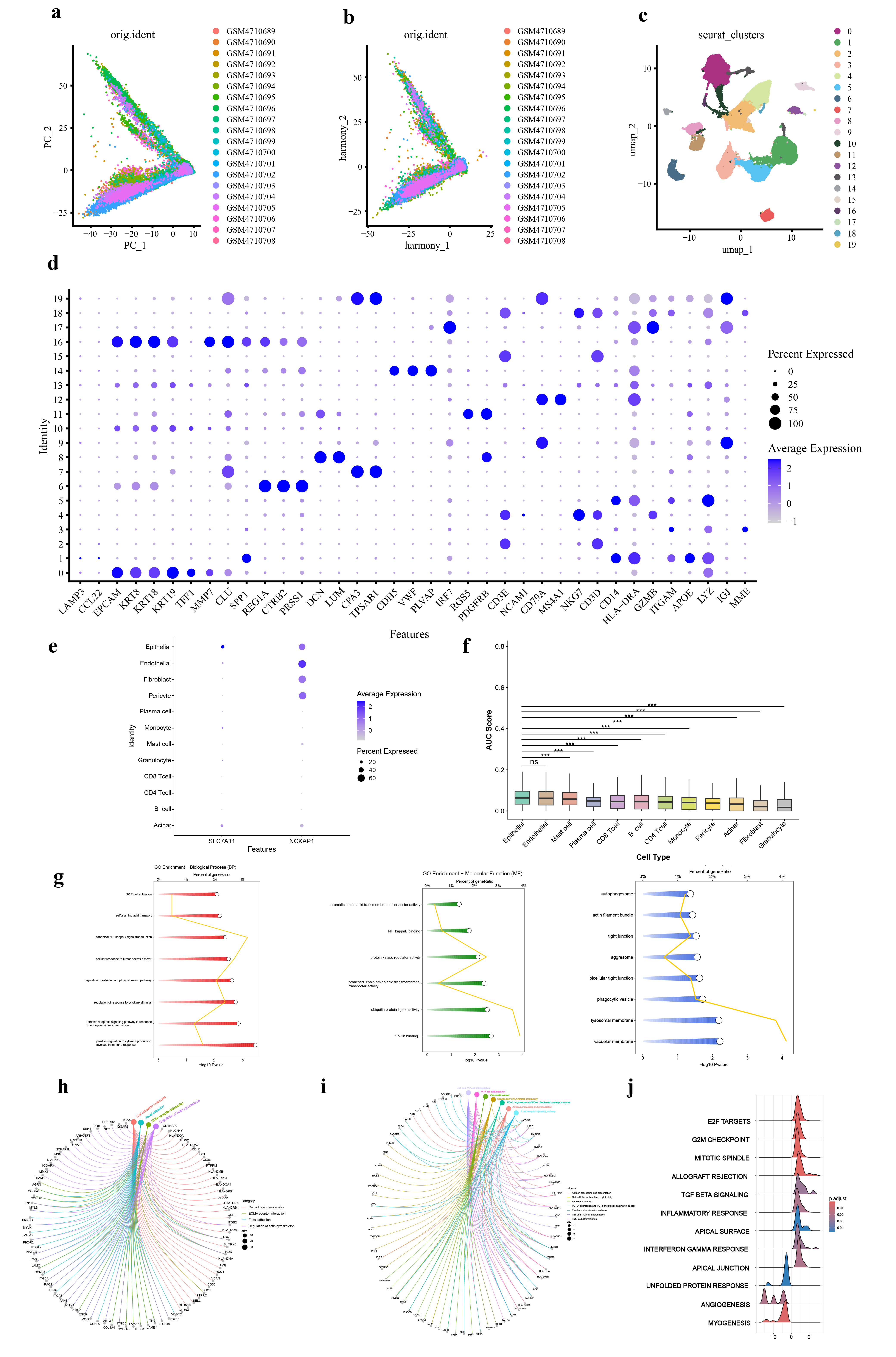

Supplement: Supplementary Figure 8 — (A, B) Dimensionality reduction distribution of single cells before and after batch effect removal. (C) All cells grouped into 20 clusters. (D) Expression of marker genes for each cluster. (E) Bubble plots of SLC7A11 and NCKAP1 expression. (F) Comparison of disulfidptosis scores across cell subsets. (G) GO enrichment analysis of DEGs between high and low disulfidptosis score groups. (H, I) KEGG functional enrichment analyses related to disulfidptosis (H) and tumorigenesis/immune pathways (I). (J) GSEA results. [file Image8.tif]

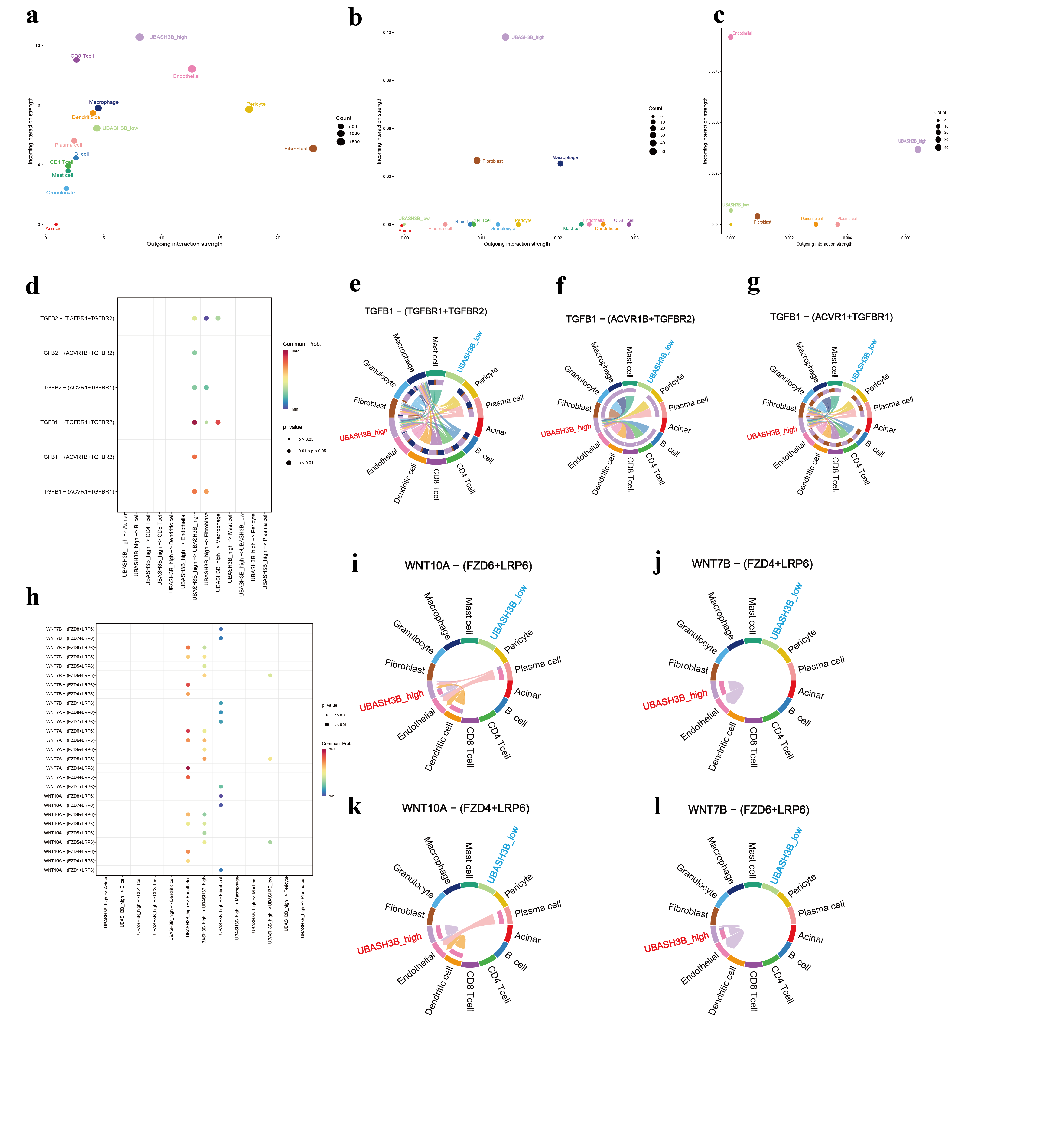

Supplement: Supplementary Figure 9 — (A) The importance of the role of individual cell subtypes in cellular communication. (B, C) The importance of the role of individual cell subtypes in TGF-β signal pathway (B) and Wnt signal pathway (C). (D) TGF-β signaling-related ligand–receptor interaction probability bubble plot between UBASH3B and various cell subpopulations. (E–G) Cross-talk of TGF-β signaling-related key ligand–receptor pairs across distinct cell populations. (H) Wnt signaling-related ligand–receptor interaction probability bubble plot between UBASH3B and various cell subpopulations. (I-L) Cross-talk of Wnt signaling-related key ligand–receptor pairs across distinct cell populations. [file Image9.tif]

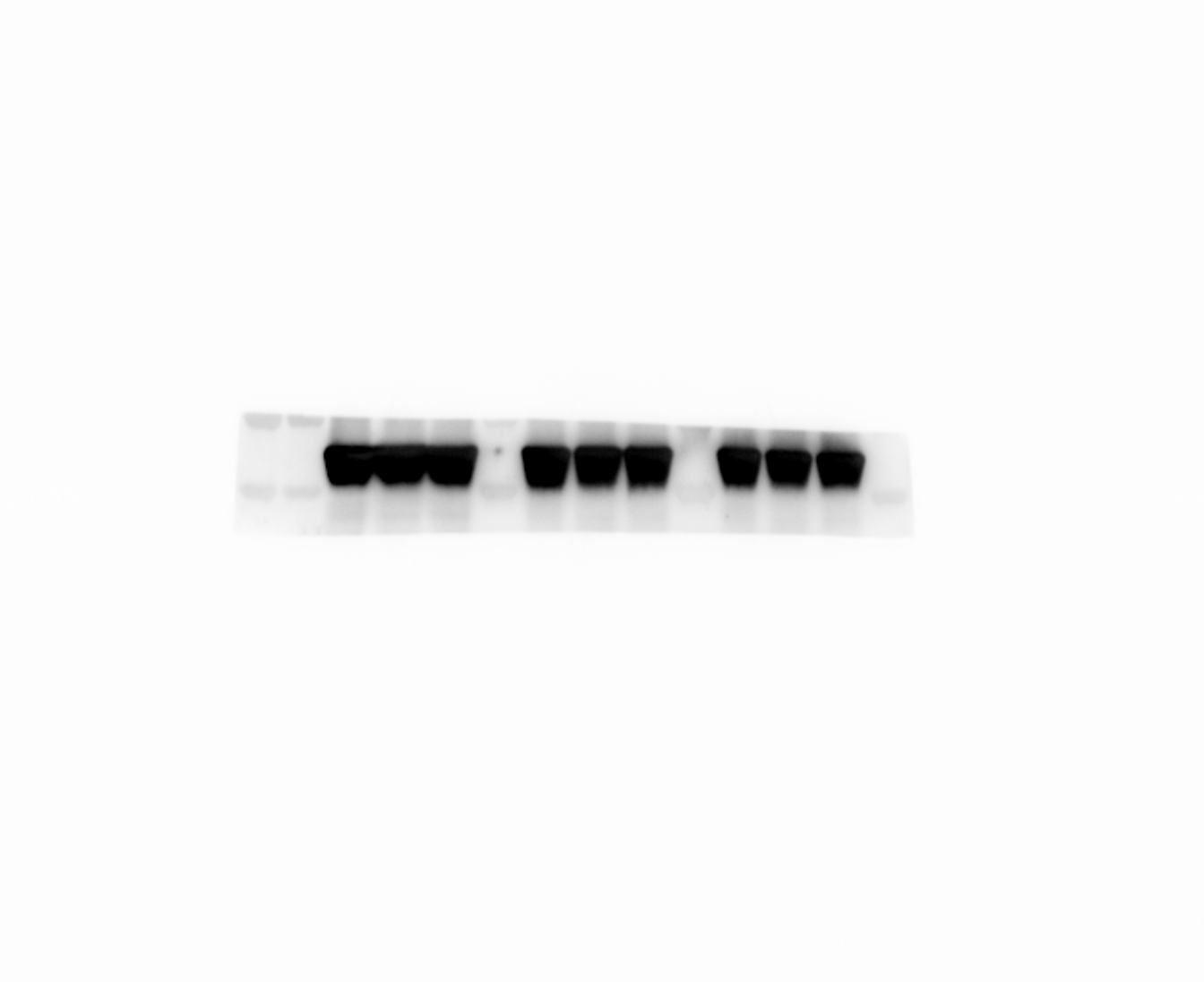

Supplement: Supplementary file 10 [file Image10.tif]

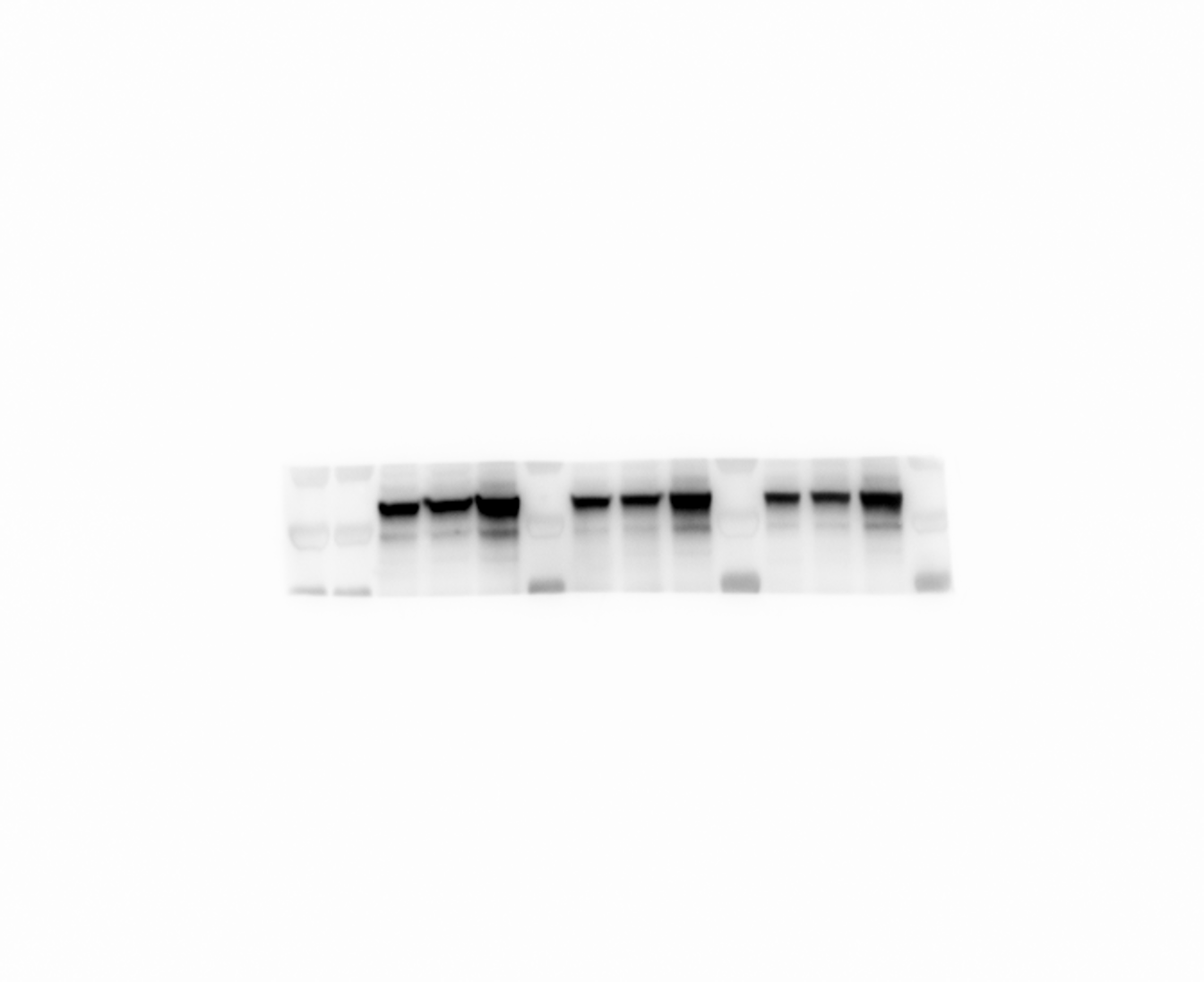

Supplement: Supplementary file 11 [file Image11.tif]

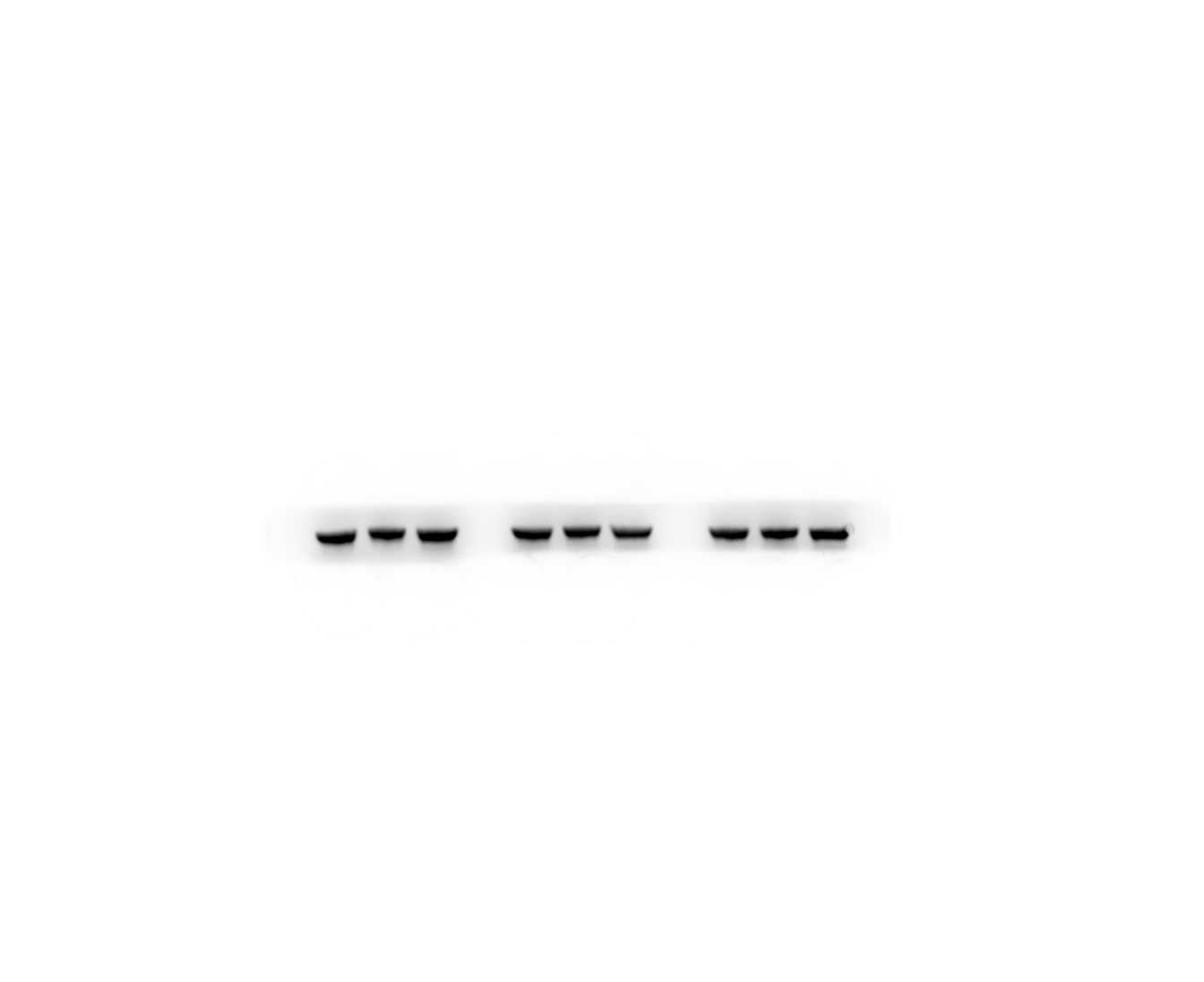

Supplement: Supplementary file 12 [file Image12.tif]

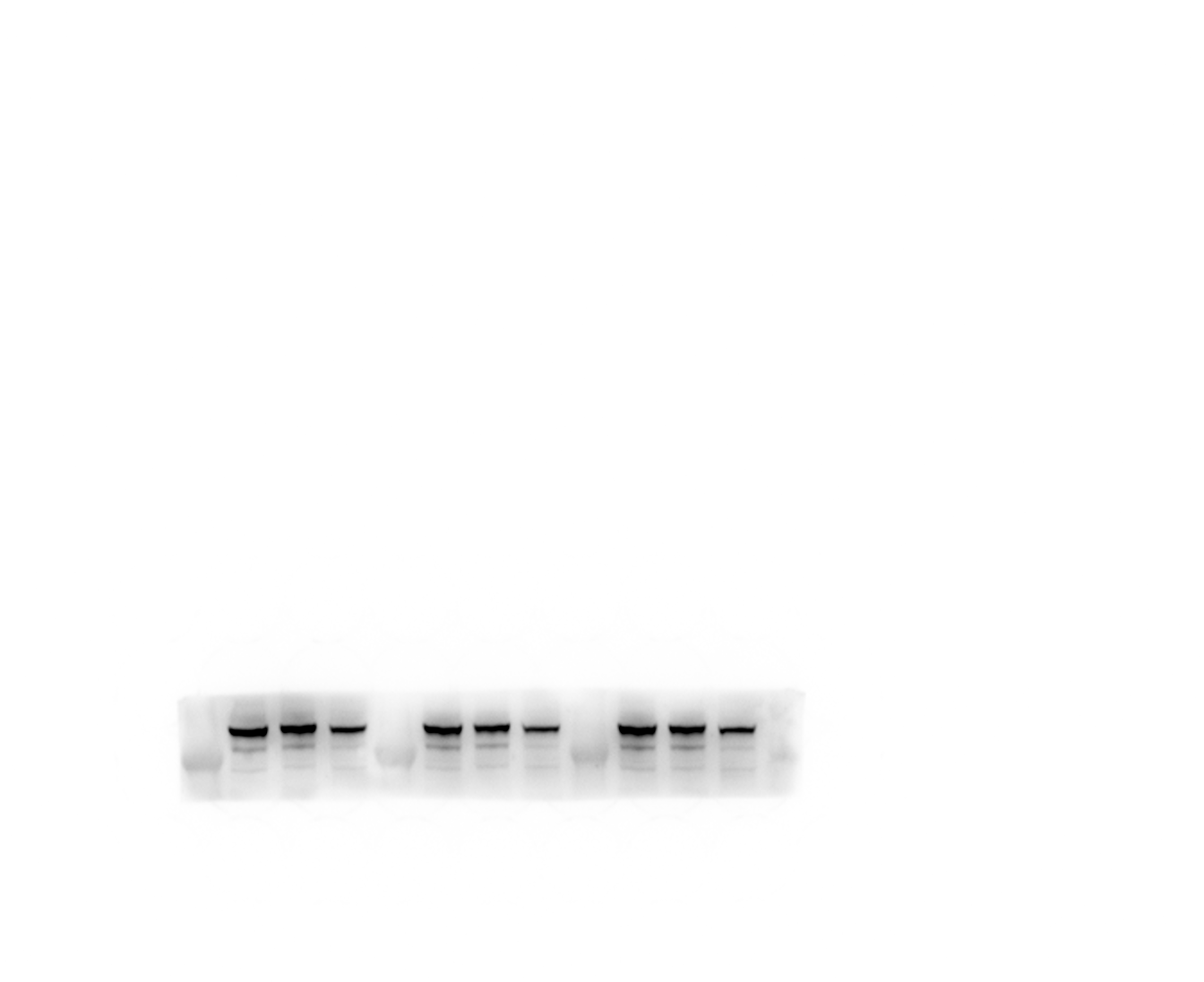

Supplement: Supplementary file 13 [file Image13.tif]
